# Early Cytokine Induction Upon *Pseudomonas aeruginosa* Infection in Murine Precision Cut Lung Slices Depends on Sensing of Bacterial Viability

**DOI:** 10.3389/fimmu.2020.598636

**Published:** 2020-10-30

**Authors:** Ulrike Kolbe, Buqing Yi, Tanja Poth, Amy Saunders, Sébastien Boutin, Alexander H. Dalpke

**Affiliations:** ^1^Department of Infectious Diseases, Medical Microbiology and Hygiene, Heidelberg University Hospital, Heidelberg, Germany; ^2^Institute of Medical Microbiology and Hygiene, Technische Universität Dresden, Dresden, Germany; ^3^CMCP—Center for Model System and Comparative Pathology, Institute of Pathology, Heidelberg University Hospital, Heidelberg, Germany; ^4^Division of Infection, Immunity and Respiratory Medicine, School of Biological Science, Faculty of Biology, Medicine and Health, Lydia Becker Institute of Immunology and Inflammation, University of Manchester, Manchester Academic Health Science Centre, Manchester, United Kingdom; ^5^Translational Lung Research Center Heidelberg (TLRC), German Center for Lung Research (DZL), University of Heidelberg, Heidelberg, Germany

**Keywords:** precision cut lung slice, *Pseudomonas aeruginosa*, live/dead discrimination, innate immunity, infection and immunity

## Abstract

Breathing allows a multitude of airborne microbes and microbial compounds to access the lung. Constant exposure of the pulmonary microenvironment to immunogenic particles illustrates the need for proper control mechanisms ensuring the differentiation between threatening and harmless encounters. Discrimination between live and dead bacteria has been suggested to be such a mechanism. In this study, we performed infection studies of murine precision cut lung slices (PCLS) with live or heat-killed *P. aeruginosa*, in order to investigate the role of viability for induction of an innate immune response. We demonstrate that PCLS induce a robust transcriptomic rewiring upon infection with live but not heat-killed *P. aeruginosa*. Using mutants of the *P. aeruginosa* clinical isolate CHA, we show that the viability status of *P. aeruginosa* is assessed in PCLS by TLR5-independent sensing of flagellin and recognition of the type three secretion system. We further demonstrate that enhanced cytokine expression towards live *P. aeruginosa* is mediated by uptake of viable but not heat-killed bacteria. Finally, by using a combined approach of receptor blockage and genetically modified PCLS we report a redundant involvement of MARCO and CD200R1 in the uptake of live *P. aeruginosa* in PCLS. Altogether, our results show that PCLS adapt the extent of cytokine expression to the viability status of *P. aeruginosa* by specifically internalizing live bacteria.

## Introduction

At the onset of infection, host cell defense depends on the rapid initiation of an innate immune response based on the recognition of a broad variety of pathogen associated molecular patterns (PAMPs) *via* pattern recognition receptors (PRRs) on innate immune cells (1, 2). In the fragile lung, balancing immune recognition with tolerance is important in light of constant exposure to airborne microbial PAMPs. Failure to regulate this balance might lead to inflammation-mediated tissue destruction or in pathogen-mediated toxicity, both resulting in organ failure (3, 4). Discriminating between live and dead microorganisms by sensing so-called viability-associated PAMPs (vita-PAMPs) has been reported to be a mechanism that allows assessing the threat of a microbial encounter (5–7).

*Pseudomonas aeruginosa* is an opportunistic gram-negative bacterium causing acute and chronic airway infections especially in immunocompromised individuals, people suffering from certain genetic disorders, or in ventilated intensive care unit patients (8). Typical PAMPs that characterize *P. aeruginosa* are flagellin and LPS that engage with TLR5 and TLR4, respectively, thereby initiating a crucial yet redundant pro-inflammatory signaling cascade (9–11). Extracellular detection of *P. aeruginosa* by plasma-membrane bound PRRs is complemented by internalization and intracellular degradation of *P. aeruginosa* (12–14). Intracellular defense against *P. aeruginosa* is for instance provided by the cytoplasmic PRR Nod1, which senses specific peptidoglycan motifs (15). However, sensing of PAMPs might not be sufficient to assess the threat coming from a versatile bacterium such as *P. aeruginosa*, whose virulence is many-faceted and may unfold *via* its five secretion systems (16). Most of the toxic mediators and enzymes, however, are secreted by the type two and the type three secretion system (T2SS and T3SS, respectively). The T2SS secretes effector molecules such as exotoxin A, elastases, and lipases directly in the extracellular space whereas the T3SS enables the bacterium to directly inject the exotoxins U, S, T, and Y into the host cell cytoplasm making the latter a powerful infectious weapon (16–18). Upon host cell contact or calcium-depletion, *P. aeruginosa* transcribes and assembles the T3SS needle complex and the translocation pore (19–21). Another important virulence factor of *P. aeruginosa* is the flagellum that enables the bacterium to swim (22). Both, the T3SS and the flagellum represent structural features assigned to live bacteria that may be employed to discriminate between live and dead *Pseudomonas* encounter.

In the current study, we used precision cut lung slices (PCLS) as a method of choice to investigate the innate immune response towards *P. aeruginosa* as a complex interplay between different pulmonary cells within one PCLS rather than looking at one cell type. Our data demonstrate that PCLS discriminate between live and dead *Pseudomonas* encounter. We show that only viable but not dead *P. aeruginosa* triggers a profound early pro-inflammatory cytokine response in PCLS. Furthermore, we were able to attribute the lack of recognition to the flagellum and the T3SS, especially to the needle protein pscF, which are functional characteristics of viable bacteria. Furthermore, internalization of bacteria, with contribution of the receptors CD200R1 and MARCO, is important for live/dead discrimination.

## Materials and Methods

### *P. aeruginosa* Strains

Experiments shown in this paper were conducted with the *P. aeruginosa* strains listed in **Table 1**. The patient isolates PA8 and PA24 were from the Diagnostic Department of the Institute for Infectious Diseases, Medical Microbiology, University Hospital Heidelberg. *Pseudomonas* cultures were grown in lysogeny broth (LB) overnight at 37°C with shaking at 200 rpm. Overnight cultures were harvested, resuspended in PBS and adjusted to McFarland 0.5 (referred to as live bacterial suspension). An aliquot of the live bacterial suspension was subsequently heat-killed by exposure to 85°C for 5 min (referred to as heat-killed bacterial suspension). For chemical fixation, 750 µl of the live bacterial suspension were pelleted, supernatant was discarded, and the pellet was resuspended in 750 µl of a 4% formaldehyde solution (PFA). Upon a 10 min incubation period at room temperature, bacteria were washed three times by centrifugation at 13,000 g for 5 min and the final pellet was resuspended in 750 µl of PBS (referred to as PFA-fixed bacterial suspension). The death of *P. aeruginosa* after heat- or PFA-treatment was controlled by plating aliquots on Columbia agar plates supplemented with 5% sheep blood (BD Biosciences, Heidelberg, Germany). Plates were checked for growth after 24 h of incubation at 37°C, 5% CO_2_ and 95% humidity.

**Table 1:** Overview of the *P. aeruginosa* strains used in this study and their origin.

No.	Name	Description	Reference
1	PAO1	Wild type *P. aeruginosa* lab strain	DSMZ 22644
2	PA8	Non-mucoid, late/chronic cystic fibrosis-isolate	This study
3	PA24	Non-mucoid, early/intermittent cystic fibrosis-isolate	This study
4	CHA	Mucoid cystic fibrosis patient isolate	(23, 24)
5	CHA ΔExoS	CHA, gentamicin cassette inserted within *exoS*	(25)
6	CHA ΔBD	CHA, gentamicin cassette inserted within *popB* and *popD*	(26)
7	CHA ΔpscF	CHA, internal deletion of the *pscF* gene	(27)
8	CHA Δflic	CHA, gentamicin cassette inserted within *fliC*	(27)
9	CHA ΔpscFΔflic	CHA, gentamicin cassette inserted within *fliC* and internal deletion of the *pscF* gene	(27)

Strains have been characterized before in the respective references.

### Mice

Wild type (WT), B6.129S1-*Tlr5*^tm1Flv^/J, and CD200R1 KO mice were on a C57BL/6 background. B6.129S1-*Tlr5*^tm1Flv^/J (TLR5 KO, JAX stock no. #008377) have previously been described (28) and were kindly provided by Prof. Dr. M. W. Hornef (Institute for Medical Microbiology, RWTH University Hospital Aachen, Aachen, Germany). CD200R1 KO mice were kindly provided by Dr. A. Saunders (Lydia Becker Institute of Immunology and Inflammation, Division of Infection, Immunity and Respiratory Medicine, School of Biological Science, Faculty of Biology, Medicine and Health, University of Manchester, Manchester Academic Health Science Centre, Manchester M13 9PL, UK) and have been described previously (29). C57Bl/6J and C57Bl/6N control mice were bought from Janvier Labs or Charles River Laboratories, respectively. Mice were housed and bred under specific pathogen-free (SPF) conditions in the animal facility of the University Heidelberg.

### Generation of Precision Cut Lung Slices

Mice were sacrificed by intraperitoneal injection with 240 mg/kg ketamine (Bremer Pharma, Warburg, Germany) and 32 mg/kg xylazine (CP-Pharma, Burgdorf, Germany) in 0.9% sodium chloride solution. Subsequently, abdomen was opened, the inferior vena cava was cut for exsanguination and the diaphragm was punctured to get access to the lungs. The pulmonary blood vessels were rinsed with PBS by puncturing the heart. After exposure of the trachea, a small incision was made and a 20G-needle with a polyethylene-tubing at its tip was carefully inserted. The lungs were subsequently inflated with 0.035 ml/g bodyweight of a 20% gelatin suspension in PBS. To quickly solidify the gelatin, the mouse was kept at 4°C for approximately 10 min. Afterwards, the left lung lobe was excised, rinsed with PBS, and gelatin-embedded in a cubic mold on ice. Upon solidification, lungs were cut transversely into 250 µm slices using a Leica VT1200S (Wetzlar, Germany) or Thermo Scientific Microm HM650V (Waltham, MA, USA). Collection of PCLS was started after discarding the top quarter of the lobe. In parallel to the cutting process, PCLS were trimmed of most of the surrounding gelatin and placed into a 24-well plate in DMEM/F12 containing 15 mM HEPES, 2.5 mM L-glutamine (Thermo Fisher Scientific, Waltham, MA, USA), and 1% Penicillin/Streptomycin on ice. Two randomly chosen PCLS were cultured per well. After completion of the cutting, PCLS were transferred to a sterile 24-well plate containing 500 µl DMEM/F12 supplemented with 1% Penicillin/Streptomycin and maintained at 37°C, 5% CO_2_, and 95% humidity. The medium was exchanged over 3 h to prevent contaminations and to remove the resolved gelatin. Generation of PCLS was considered to be completed afterwards. Experiments were done according to the local animal welfare rules and upon notification of the local authorities.

### Imaging of Precision Cut Lung Slices

Brightfield microscopic images of PCLS were taken with the ECHO Rebel Hybrid Microscope (Discover-ECHO, San Diego, CA, USA). For histopathological evaluation of selected PCLS, tissue preparation was carried out at the Center for Model System and Comparative Pathology, Institute of Pathology, University Hospital Heidelberg. Briefly, after overnight fixation in 10% buffered formalin, representative specimens were routinely dehydrated, embedded in paraffin, and then cut into 4 μm-thick sections. The tissue sections were stained with H&E. Additionally, a periodic acid-Schiff (PAS) reaction was performed. Scans of H&E stained PCLS tissue sections were prepared by the Tissue Bank of the National Center for Tumor Diseases (NCT) Heidelberg, Germany. Representative images of the scans were made using the software QuPath version 0.2.1 (30). Scale bars shown were added with the software Fiji version 1.52p (31).

### Infection of Precision Cut Lung Slices

Infection of PCLS was carried out 1 day post cutting. If not stated otherwise, the overnight incubation medium was refreshed by 300 µl DMEM/F12 the next morning before conducting experiments. PCLS were infected with 10 µl of the live, heat-killed, or PFA-fixed bacterial suspension. For classical PAMP stimulation, PCLS were treated with 5 µg/ml ultrapure *Pseudomonas aeruginosa-*derived flagellin (InvivoGen, San Diego, CA, USA) or 0.1 µg/ml ultrapure LPS derived from *Salmonella minnesota* R595 (InvivoGen, San Diego, CA, USA). Heat-treatment of flagellin and LPS was carried out at 85°C for 5 min before infection. For intracellular delivery of flagellin, the stimulus was packaged with the Pierce Protein Transfection Reagent Kit (Thermo Fisher Scientific, Waltham, MA, USA) according to the manufacturer’s instructions. Four hours post infection, PCLS were either subjected to RNA isolation or 100 µg/ml ciprofloxacin was added per well before further incubation. Protein isolation and collection of conditioned supernatants was conducted 6 h post infection. For antibody-mediated receptor blockage, PCLS were incubated with 10 µg/ml monoclonal MARCO antibody (Bio-Rad, Hercules, CA, USA) or 10 µg/ml of mouse IgG1 isotype control (Thermo Fisher Scientific, Waltham, MA, USA) 60 min prior to infection. For inhibition of phagocytosis, 5 µM Cytochalasin D (from *Zygosporium mansonii*, Sigma-Aldrich, Saint Louis, MO, USA) was added 30 min prior to infection.

### RNA Isolation and Quantitative RT-PCR

PCLS tissue was mechanically lysed in tubes filled with glass beads (diameter 0.1 mm [Carl Roth, Karlsruhe, Germany]) and 350 µl RLT lysis buffer (Qiagen, Hilden, Germany) supplemented with 1% β-mercaptoethanol for 40 s using a Mini-Bead-Beater (BioSpec Products Inc., Bartlesville, OK, USA). Total RNA was prepared from the supernatant according to the instructions of the RNeasy Mini Kit (Qiagen, Hilden, Germany). The DNase treatment was performed using the RNase-free DNase set (Qiagen, Hilden, Germany). RNA was reverse transcribed into cDNA using the High-Capacity cDNA Reverse Transcription Kit (Applied Biosystems, Waltham, MA, USA) with a minimum of 50 ng of RNA input depending on the biological replicate. Gene expression analysis was done by qRT-PCR in a 96-well format using 1:4-diluted cDNA, the Fast SYBR Green Master Mix (Applied Biosystems, Waltham, MA, USA) and corresponding primers. Amplification was conducted in a 40 cycles loop (95°C for 3 s, 60°C for 30 s) on a StepOnePlus Real-Time PCR System (Applied Biosystems, Waltham, MA, USA). The C_t_ value of the housekeeping gene (*Gapdh*) was subtracted from the C_t_ values of the gene of interest for a given sample. Afterwards, the ΔC_t_ value of the non-treated control was subtracted from the ΔC_t_ value of a certain experimental condition yielding the corresponding ΔΔC_t_ value. The fold change was calculated using the formula 2^-ΔΔCt^. The following primer sequences were used for quantitative real-time PCR: mouse GAPDH, forward, 5´-TTCACCACCATGGAGAAGGC-3´ and reverse, 5´-GGCATGGACTGTGGTCATGA-3´; mouse KC, forward, 5´-CCGAAGTCATAGCCACACTC-3´ and reverse, 5´-GTCAGAAGCCAGCGTTCAC-3´; mouse IL6, forward, 5´-CTGCAAGAGACTTCCATCCAG-3´ and reverse, 5´-AGTGGTATAGACAGGTCTGTTGG-3´; mouse MIP-2, forward, 5´- AGACAGAAGTCATAGCCACTCTCAAG -3´ and reverse, 5´-CCTCCTTTCCAGGTCAGTTAGC -3´; mouse MARCO, forward, 5´-ACCAGGCCTACCAGGTTTG-3´ and reverse, 5´-ACCCTGCACTCCAGGTTTT-3´; mouse CD200R1, forward, 5´-TACAAAGGCTCTGCTCTGCT-3´ and reverse, 5´-AAGCAGCTGGTTTCATTGGT-3´

### Protein Isolation and BCA Assay

PCLS were transferred into a tube filled with glass-beads and 150 µl RIPA lysis buffer (50 mM Tris-HCl pH 7.4, 150 mM NaCl, 1 mM EDTA, 1% NP-40, 0.25% sodium deoxycholate) supplemented with protease and phosphatase inhibitor cocktails (Roche, Basel, Switzerland). The PCLS were mechanically lysed by beat beating for 10 s. After incubation at 4°C for 20 min, supernatant was harvested upon centrifugation at 4°C and 14,000 g for 20 min. The BCA assay was performed in half-area ELISA plates following the instructions of the Pierce BCA Protein Assay Kit (Thermo Fisher Scientific, Waltham, MA, USA). Protein levels were quantified by measuring the absorbance at 570 nm using the SUNRISE absorbance reader (Tecan, Salzburg, Austria) and analyzed with the Magellan V 5.0 software (Tecan, Salzburg, Austria).

### Measurement of Cytokine Secretion

Quantification of IL-6 (BD Biosciences, Heidelberg, Germany) content in supernatant harvested from PCLS was done by ELISA according to the manufacturer’s instructions. Quantification of cytokine levels was done by measuring the absorbance at 450 and 570 nm as a reference using the TECAN SUNRISE Absorbance reader (Tecan, Salzburg, Austria) and analyzed with the Magellan V 5.0 software (Tecan, Salzburg, Austria). The CBA-assay LEGENDplex Mouse Inflammation Panel (BioLegend, San Diego, CA, USA) was used to assess cytokine levels in conditioned supernatant according to the manufacturer’s instructions. To correct for size differences between individual PCLS, the amount of detected cytokine was normalized to the protein concentration originating from the lysis of the corresponding PCLS tissue.

### MTS Assay

The CellTiter 96 Aqueous One Solution Cell Proliferation Assay (Promega, Madison, WI, USA) was used to assess metabolic activity of the PCLS as a readout for viability. Two hours post infection of equally sized PCLS, 60 µl of the MTS tetrazolium compound were added to each condition. Additionally, the MTS reagent was added to a PCLS maintained in 1% TritonX-100 as a negative control. The infected PCLS were incubated for further 2 h in the presence of the MTS tetrazolium compound. Four hours post infection, the purple formazan product was dissolved in culture medium by shaking the 24-well plate for 1 min. Subsequently, 100 µl of the supernatant was transferred in duplicates into a 96-well plate and the absorbance was measured at 492 nm using the TECAN SUNRISE Absorbance reader (Tecan, Salzburg, Austria) and analyzed with the Magellan V 5.0 software (Tecan, Salzburg, Austria).

### Lactate Dehydrogenase Assay

Lactate Dehydrogenase (LDH) assay was performed with the Pierce™ LDH Cytotoxicity Assay Kit (Thermo Fisher Scientific, Waltham, MA, USA). After completing the PCLS generation, supernatant of PCLS was harvested in 24 h-intervals over 2 days and replaced with fresh 500 µl DMEM/F12 supplemented with 1% Penicillin-Streptomycin between two 24-h intervals. As positive control for LDH release, PCLS were treated with 1% TritonX-100 at 37°C, 5% CO_2_, and 95% humidity for 45 min. The LDH assay was performed immediately after harvesting the supernatant according to the manufacturer’s instructions. The absorbance was measured at 492 nm and at a reference wavelength of 620 nm with the TECAN SUNRISE Absorbance reader (Tecan, Salzburg, Austria) and the Magellan V 5.0 software (Tecan, Salzburg, Austria).

### Transcriptome Analysis of Infected Precision Cut Lung Slices

For expression profiling, RNA was isolated with the RNeasy Mini Kit (Qiagen, Hilden, Germany) from non-treated PCLS, PCLS infected with live strain CHA, and PCLS infected with heat-killed strain CHA 4 h post infection. The quality of the RNA was analyzed using the Agilent Bioanalyzer system following the instructions of the RNA 6000 Nano Kit (Agilent Technologies, Santa Clara, CA, USA). The RNA integrity number (RIN) of all RNA preparations was at least nine. RNA samples were sequenced by the DRESDEN-concept Genome Center at the Center for Molecular and Cellular Bioengineering (CMCB), Technische Universität Dresden, Dresden, Germany. Total RNA was enriched for mRNA species by poly-dT pulldown according to the instructions of the NEBNext Poly(A) mRNA Magnetic Isolation Module (New England Biolabs, Ipswich, MA, USA). Libraries were prepared using the NEBNext Ultra II Directional RNA Library Prep Kit for Illumina (New England Biolabs, Ipswich, MA, USA) according to the manufacturer’s protocol. Sequencing was performed on a NextSeq500 (Illumina Inc., San Diego, CA, USA) in single-end mode (75 bp). Per sample, around 30 million fragments were sequenced. Resulting sequences were trimmed for quality using Sickle 1.33 (parameters, quality >30; length>45) (32). Reads were mapped to the *Mus musculus* genome assembly *GRCm38* (mm10) using Hisat2 (v2.1.0) (33), alignment was sorted using samtools (v1.9) (34). Transcripts were annotated and quantified with Stringtie (v1.3.6) (35). For the differential analysis, data were analyzed with R (v3.6.2) and the package Ballgown (36) and DEseq2 (37). Ballgown was used to estimate and correct the abundance of each transcript. Deseq2 was used to analyze the differential expression of each transcripts between the experiments and the control of the false discovery rate due to multiple comparison was done using the Benjamini & Yekutieli method (38). Raw sequences data and script are available publicly (SAMEA7231371-79).

Functional annotation of gene sets was performed using the R package clusterProfiler (39) (version 3.16.0) to find enriched pathways. Gene Ontology Enrichment Analysis (GOEA) was conducted using the GO biological process terms. Genes showing differential expression (|FC| ≥ 1.5, adjusted p-value ≤ 0.05) were used as input for the analysis. R package ggplot2 (version 3.3.0) was used to visualize the data of normalized counts generated by DESeq2.

### Assessment of Viable Bacteria

A live bacterial suspension was generated in PBS supplemented with 0.1% Tween. The viable bacteria were assessed as described in the manufacturer’s manual QUANTOM™ Viable Cell Staining Kit (Logos Biosystems, Anyang, South Korea). In short, 10 µl of the McFarland 0.5 bacterial suspension were incubated with 2 µl of the Viable Cell Staining Dye, a Calcein AM derivative. The mixture was incubated for 1 h at 37°C in the dark. Afterwards, 8 µl of the Cell Loading Buffer I was added and 6 µl of the resulting mixture was loaded on a M50 Cell Counting Slide. Upon centrifugation at 300 RCF for 30 min in a QUANTOM™ Centrifuge to sediment the bacteria, the fluorescence (Ex. 496 nm, Em. 520 nm) of the viable bacteria was measured using the QUANTOM Tx Microbial Cell Counter (Logos Biosystems, Anyang, South Korea).

### Statistical Analysis

All experiments were performed with at least three independent biological replicates. The exact number of repetitions is stated in each graphical illustration (n). For comparisons between more than two groups but one independent variable, the One-way-ANOVA was performed with matching for the biological replicates when applicable and with Sidak as post-test to correct for multiple comparisons. In case of two independent variables, statistical significance was assessed using the Two-way ANOVA with matching for the biological replicates when applicable and with Sidak as post-test to correct for multiple comparison. A ratio paired t-test was used to compare two groups with experimental variation between biological replicates. Results were labeled as being significantly different according to the following guidelines: *p < 0.05; **p < 0.01; ***p < 0.001. Graphical visualization and statistical analysis were performed with GraphPad Prism version 6.07 for Windows (GraphPad Software, San Diego, CA, USA, www.graphpad.com).

## Results

### PCLS Represent Small Functional Pulmonary Units

**Figure 1:**
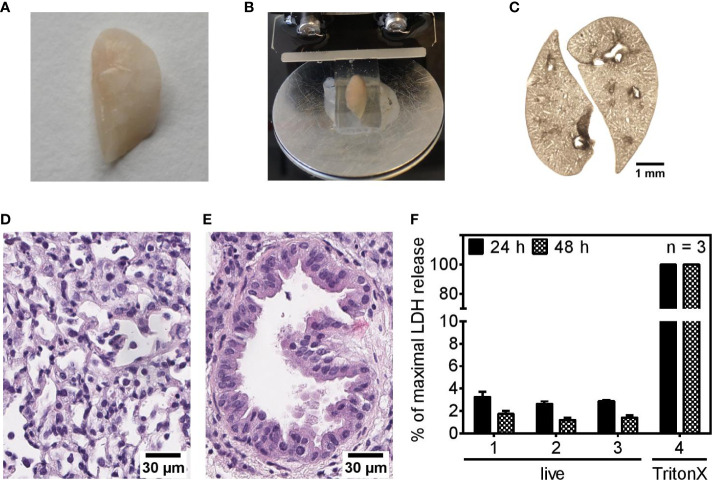
Precision cut lung slices show typical pulmonary features and remain viable in cell culture. **(A–F)** Murine lungs were inflated with a gelatin solution **(A)**, embedded in gelatin, and cut into 250 µm slices **(B)**. A brightfield image shows 1-day-old PCLS cultured under submerged conditions **(C)**. **(D, E)** Representative images of H&E-stained pulmonary tissue **(D)** and a bronchiole **(E)** of 24-h-old PCLS. **(F)** Release of lactate dehydrogenase (LDH) into the culture medium of PCLS was assessed 24- and 48 h after completing the PCLS generation process. For maximal LDH release, PCLS were treated with 1% TritonX-100 to disrupt plasma membrane integrity and trigger LDH release into the supernatant. Values are given as percentage of maximal LDH release. Shown are mean data (± SEM) of three independent experiments. Analysis was performed with three technical replicates (1–3) to achieve greater sampling. Each technical replicate encompassed two PCLS per well.

This study was conducted with murine precision cut lung slices (PCLS) as an *ex vivo* lung model system to investigate the regulation of immune responses as a complex interplay between pulmonary cells organized in their natural architecture rather than studying an isolated pulmonary cell type. For PCLS generation, gelatin-inflated lungs of euthanized mice were isolated, the left lobe was separated (**Figure 1A**), gelatin-embedded, and cut into thin slices, PCLS (**Figure 1B**). A representative brightfield microscopy image shows the optical appearances of 1-day-old PCLS maintained in a submerged cell culture system (**Figure 1C**). The two-fold magnification used for taking the brightfield image of the PCLS allows recognition of the fine structure of hollow cavities pervading the lung tissue representing the bronchiolar system branching off the main left bronchus. Pulmonary architecture of cultured PCLS was further analyzed by hematoxylin and eosin (H&E) staining, revealing typical features such as lung parenchyma spaced by airways (**Figure 1D**) and bronchial epithelial cells with cilia outlining a bronchiole (**Figure 1E**). Of note, cellular lesions and autolysis occur to some extent and most probably trace back to unavoidable artifacts during preparation of H&E staining which involves physical handling and thus manipulation of the PCLS, which, under experimental conditions, is avoided once PCLS are taken into culture. PAS staining revealed no cross-contamination with fungi or bacteria of naïve PCLS (not shown). To further address the degree of cutting-related cellular disruption and to estimate the viability of the cells that comprise the PCLS, we measured the release of the cytoplasmic enzyme lactate dehydrogenase (LDH) into the PCLS culture medium. Release of LDH was very low for both, 24- and 48-h-old PCLS compared to PCLS treated with TritonX-100 indicating good viability of the slices (**Figure 1F**). Knowing that one-day old PCLS stay viable and exhibit typical pulmonary features, we set out to analyze their response to live and dead *P. aeruginosa*.

### PCLS Discriminate Between Live and Dead *Pseudomonas aeruginosa* Infection

Live/dead discrimination was examined using the wild type lab strain PAO1 and the cystic fibrosis isolates PA8 and PA24. For infection with dead *P. aeruginosa*, the strains were heat-killed and subsequently plated on blood agar plates to verify killing of the bacteria. To prevent continuous growth of the live bacteria whilst the killing procedure was in progress, the heat-killing was defined as a 5 min procedure and 85°C were chosen after preliminary experiments to ensure killing of bacteria within these 5 min. Hence, PCLS were exposed to roughly 3.64 × 10^5^ live or heat-killed CFU initially (reference strain CHA), ensuring an equal starting point for both infection scenarios (**Supplementary Figure S1**).

**Figure 2:**
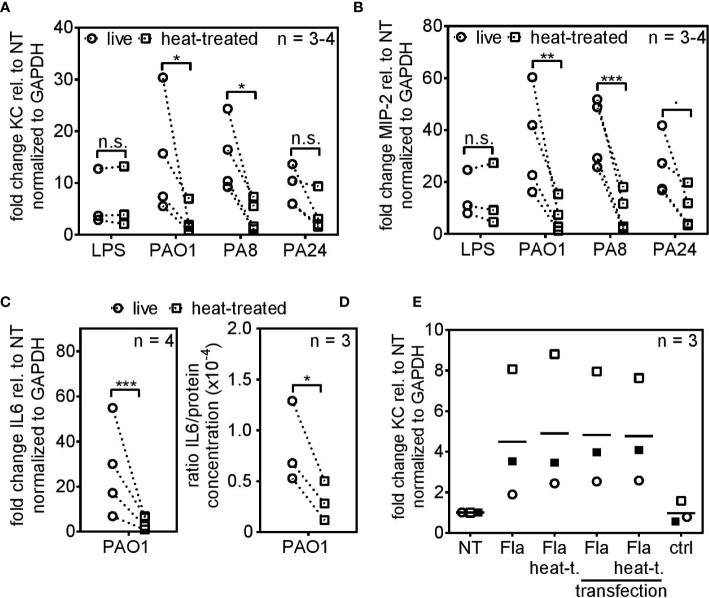
PCLS trigger robust cytokine expression in response to live but not heat-killed *Pseudomonas aeruginosa*. **(A–D)** PCLS were infected with the TLR4-ligand LPS, the lab strain *P. aeruginosa* PAO1 or the cystic fibrosis-isolates PA8 or PA24. Expression of KC **(A)**, MIP-2 **(B)**, and IL6 **(C)** were analyzed by quantitative real-time PCR 4 h post infection. *Gapdh* served as housekeeping gene. Fold change was calculated by the ΔΔC_t_ method. **(D)** PCLS were infected with PAO1 and IL6 release into the supernatant was assessed by ELISA 6 h post infection. IL6 levels were normalized to the protein concentration originating from PCLS tissue lysis. **(E)** PCLS were stimulated with flagellin (Fla), heat-treated flagellin (Fla heat-t.) or left untreated (NT). For intracellular delivery, non-treated and heat-treated flagellin was packaged and PCLS were transfected. PCLS treated with the transfection agent only served as control (ctrl). KC expression was analyzed 4 h post stimulation by quantitative real-time PCR. G*apdh* served as housekeeping gene and fold change was calculated according to the ΔΔC_t_ method. Individual experiments are displayed as symbol and lines **(A–D)** or scatter dot plot with the mean value indicated by a line **(E)**. Number of repetitive experiments is stated (n). n.s., not significant; ∙p = 0.053; *p < 0.05, **p < 0.01, ***p < 0.001.

Live PAO1 and PA8 triggered robust KC and MIP-2 expression 4 h post infection, whereas their dead equivalents did not (**Figures 2A, 2B**, respectively). Live and heat-killed PA24 showed the same trend, yet due to experimental variation did not reach statistical significance. PA24 is a clinical isolate associated with early/intermittent infection in a patient with cystic fibrosis but still produced pyocyanin and biofilm (data not shown). The delicate process of PCLS preparation as well as the random assignment of each PCLS to experimental conditions may be responsible for some experimental variation seen in the figures. To analyze whether reduced cytokine mRNA expression leads to lower cytokine secretion as well, we additionally analyzed IL6 expression and IL6 secretion 4 and 6 h post infection, respectively. We observed reduced IL6 mRNA levels (**Figure 2C**) and in parallel diminished IL6 protein levels (**Figure 2D**) in response to heat-killed bacteria. To exclude the possibility that the significant gap between cytokine induction of live and heat-killed *P. aeruginosa* originates from the destruction of the two fundamental PAMPs LPS and flagellin, both were subjected to the heat-killing procedure and PCLS were subsequently stimulated with the non-treated and heat-treated version. Both PAMPs, irrespective of heat-treatment, retained their ability to induce cytokine expression in PCLS (**Figures 2A, B, E**). Flagellin can be a potent signaling activator inside and outside of the cell. Accordingly, non-treated and heat-treated flagellin were packaged and transfected into PCLS to allow intracellular signaling independent of the surface-localized TLR5. As shown in **Figure 2E**, flagellin retained its intracellular stimulatory capacity despite heat-treatment. Hence, the substantial reduction in cytokine induction by heat-killed *P. aeruginosa* prompted us to assume that viability-associated factors of live bacteria, so-called vita-PAMPs, are essential to initiate an innate immune response in PCLS.

### Viable *P. aeruginosa* Is Recognized *via* the T3SS and Flagellin

**Figure 3:**
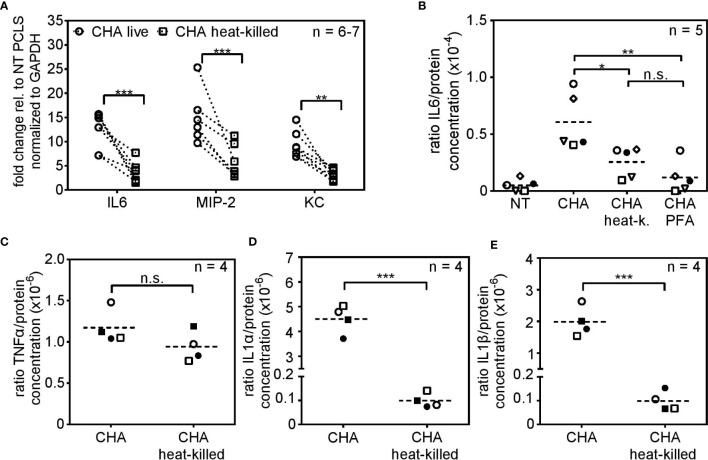
Viability-dependent cytokine induction in response to the clinical isolate CHA. **(A)** PCLS were infected with live or heat-killed CHA and IL6, MIP-2 and KC expression were analyzed by quantitative real-time PCR 4 h post infection. *Gapdh* was used as housekeeping gene. Fold change was calculated by the ΔΔC_t_ method. **(B)** PCLS were infected with live CHA, heat-killed (heat-k.) CHA or formaldehyde (PFA)-treated CHA. Release of IL6 into the supernatant was assessed by ELISA 6 h post infection. The amount of released IL6 was normalized to the protein concentration of PCLS tissue lysate. **(C–E)** Supernatant of live or heat-killed CHA-infected PCLS was analyzed with the LEGENDplex Mouse Inflammation Panel 6 h post infection. Concentration of the TNFα **(C)**, IL1α **(D)**, and IL1β **(E)** was normalized to the total protein concentration of the PCLS tissue. **(A–E)** Individual experiments are displayed as symbol and lines **(A)** or scatter dot plots with the mean value indicated by a dashed line **(B–E)**. Number of repetitive experiments is stated (n). n.s., not significant; *p < 0.05, **p < 0.01, ***p < 0.001.

**Figure 4:**
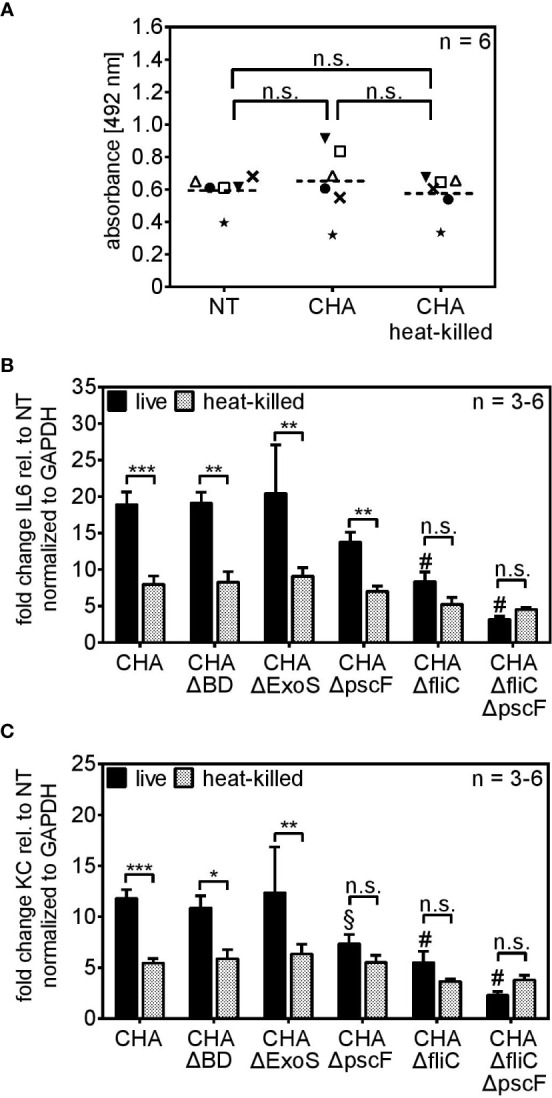
PCLS sense viable *P. aeruginosa via* the T3SS-needle protein pscF and the flagellum-monomer flagellin. **(A)** PCLS were left untreated (NT) or infected with live or heat-killed CHA. The conversion of the MTS compound into purple medium-soluble formazan was analyzed by absorbance measurement. **(B, C)** PCLS were infected with live or heat-killed CHA, CHA ΔBD, CHA ΔExoS, CHA ΔpscF, CHA ΔfliC, or CHA ΔfliCΔpscF for 4 h. IL6 **(A)** and KC **(B)** levels were quantified on RNA level by real-time PCR. Fold change of the normalized data (to *Gapdh*) was calculated with the ΔΔC_t_ method. **(A–C)** Individual experiments are displayed as scatter dot with the mean value indicated by a dashed line **(A)** or bar plots of the mean values (± SEM) **(B, C)**. Number of repetitive experiments is stated (n). n.s., not significant; *p < 0.05, **p < 0.01, ***p < 0.001. § = **p < 0.01 compared to PCLS infected with live CHA, # = ***p < 0.001 compared to PCLS infected with live CHA.

Two fundamental viability-associated features of *P. aeruginosa* are the T3SS, whose assembly is initiated upon host cell encounter (40) and the flagellar swimming motility (41). Hence, we set out to analyze the role of the T3SS and the flagellum for live/dead discrimination using the clinical CF-isolate CHA und mutated derivates thereof. As shown before for the *P. aeruginosa* strains PAO1 and PA8, infection of PCLS with heat-killed parental CHA lead to significantly reduced cytokine expression compared to infection with viable CHA 4 h post infection (**Figure 3A**). The stimulatory capacity of live CHA and live PAO1 compared to non-treated PCLS was in the same range as seen in **Supplementary Figure S2**. IL6 secretion was furthermore significantly reduced in response to heat-killed CHA 6 h post infection on protein level (**Figure 3B**). The reduction in IL6 secretion was independent of the method of bacterial inactivation as IL6 levels were also significantly reduced in response to CHA chemically fixed with 4% para-formaldehyde (PFA), confirming the importance of vita-PAMPs (**Figure 3B**). In contrast, TNFα (**Figure 3C**) and IL10 (**Supplementary Figure S3A**) secretion appeared independent of the viability status of CHA. We further found significantly increased IL1α (**Figure 3D**) and IL1β (**Figure 3E**) levels in response to live CHA compared to heat-killed CHA. Secretion of other cytokines was measured as part of the CBA-assay and results are displayed in the **Supplementary Figures S3B–D**. Of note, infection of PCLS with live CHA did not alter viability compared to PCLS infected with heat-killed CHA as shown by measuring the metabolic activity of PCLS (**Figure 4A**).

To investigate the role of the T3SS and the flagellum, we used mutants of the CF-clinical isolate CHA lacking components of both structures (**Table 1**). The CHA ΔBD does not express the pore forming units popB/D, CHA ΔpscF lacks expression of the monomer pscF that builds up the needle-like appendage of the T3SS, and CHA ΔExoS does not produce the exotoxins S which is secreted *via* the conduit of the T3SS. Additionally, we used a CHA ΔfliC mutant that does not express flagellin and thus lacks a functional flagellum and a CHA ΔfliCΔpscF double mutant that lacks both, the T3SS-needle monomer pscF and the flagellar-monomer flagellin. The CHA ΔBD and CHA ΔExoS mutants induced an IL6 (**Figure 4B**) and KC (**Figure 4C**) profile resembling the one of parental CHA. CHA ΔpscF induced significant higher IL6 expression as a live than as a heat-killed bacterium. However, in case of KC, live/dead discrimination for CHA ΔpscF was lost. Furthermore, in the absence of a functional T3SS, the KC stimulatory capacity was significantly reduced compared to live parental CHA (§ in **Figure 4C**). Furthermore, the difference in the IL6 and KC inducing activity for live *versus* dead CHA ΔfliC was lost (n.s. in **Figures 4B, C**, respectively). Of note, infection of PCLS with CHA ΔfliC lead to a significantly reduced capacity to induce IL6 and KC expression compared to parental live CHA (# in **Figures 4B, C**). Accordingly, the CHA ΔfliCΔpscF double mutant showed very weak IL6 and KC expression as live and heat-killed bacterium indicating that both monomers, pscF and flagellin, are crucial for cytokine expression in response to live *P. aeruginosa* in PCLS. However, the role of pscF as pre-dominant immunogenic structure of the T3SS seems to be cytokine-dependent. To investigate the importance of flagellin as a vita-PAMP by conferring swimming motility to the bacterium rather than as a simple monomeric ligand of the surface-localized PRR TLR5, we infected TLR5 KO PCLS with live and heat-killed CHA ΔfliC, CHA ΔpscF or CHA ΔfliCΔpscF and assessed cytokine expression. The absence of functional TLR5 did not affect the cytokine induction by live or heat-killed CHA strains compared to WT PCLS, excluding a substantial role of extracellular sensing of monomeric flagellin *via* TLR5 for live/dead discrimination (**Supplementary Figure S4**). Taken together, these results demonstrate that the T3SS, with special emphasis on pscF, along with flagellin are essential components characterizing live *P. aeruginosa* and trigger a cytokine response in PCLS.

### Profound Transcriptome Rewiring in Response to Live CHA but Not Heat-Killed CHA

**Figure 5:**
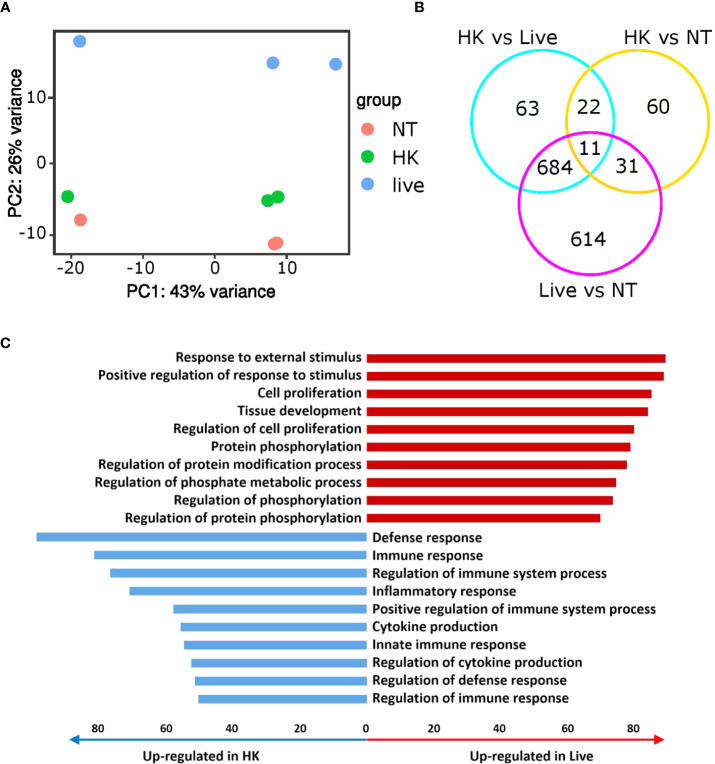
Immune responses towards live and heat-killed CHA are very distinct. **(A, B)** Non-treated (NT) PCLS and PCLS infected with live or heat-killed CHA were lysed 4 h post infection for RNA isolation and expression profiling. **(A)** PCA plot of three independent experiments. **(B)** Venn diagram showing the significantly different genes (fold change ≥2 or ≤−2 and p-value adjusted ≤ 0.05). **(C)** Ten most upregulated (red) and downregulated pathways (blue) in live CHA infected PCLS (Live) compared to heat-killed CHA (HK) treated PCLS. Bar length indicates number of significantly upregulated or downregulated genes enriched within specific pathways. HK, heat-killed CHA; live, live CHA; NT, non-treated CHA.

To further investigate the mechanisms involved in the discrimination between live and heat-killed *P. aeruginosa*, we performed RNA expression profiling comparing non-treated PCLS, PCLS infected with live or heat-killed CHA. The principal component analysis (PCA) plot shows a distinct separation of all three groups and a clear tendency of the heat-killed and non-treated samples to group together whereas the live samples show a prominent separation (**Figure 5A**). Accordingly, we found that 1,340 genes (Live *vs* NT in **Figure 5B**) were significantly regulated in PCLS upon stimulation with live CHA compared to non-treated PCLS. In contrast, heat-killed CHA significantly regulated only 124 genes (HK *vs* NT in **Figure 5B**) compared to non-treated PCLS, of which 42 genes were regulated independently of the viability status of CHA. The huge number of differentially regulated genes illustrates the viability-dependent profound rewiring of the PCLS transcriptome. Furthermore, we found that 780 genes are significantly different between PCLS infected with heat-killed and PCLS infected with live CHA (HK *vs* Live in **Figure 5B**) highlighting a considerable viability-dependent difference in the innate immune response. Pathway analysis revealed that stimulation of PCLS with live CHA promoted an inflammatory response as seen by an upregulation of pathways involved in phosphorylation as well as protein modification indicative of on-going changes in signaling cascades (**Figure 5C**). In contrast to that, infection of PCLS with heat-killed CHA favored general regulatory pathways. Individual genes grouping into each GO-term depicted in **Figure 5C** are listed in the **Supplementary Tables T1** and **T2**, respectively.

**Figure 6:**
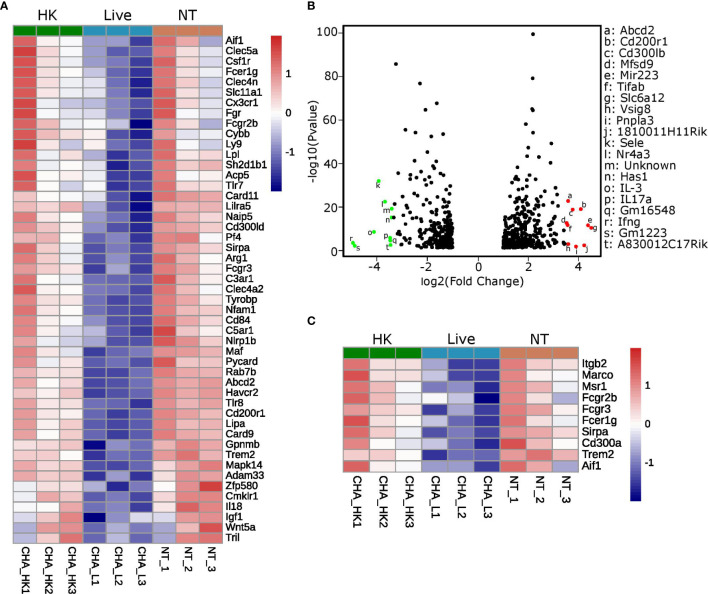
MARCO and CD200R1 are identified as candidates regulating cytokine induction in PCLS. **(A–C)** Expression profiling was performed with non-treated PCLS (NT), PCLS infected with live CHA (live) and PCLS infected with heat-killed CHA (HK). **(A)** Signaling pathway analysis was performed with genes commonly downregulated in the comparisons “live vs. NT” and “live vs. HK” (fold change ≤−1.5 and p-value adjusted ≤0.05). Heatmap shows the genes grouping into the GO term “cytokine production.” HK, heat-killed CHA; live, live CHA; NT, non-treated CHA. **(B)** Volcano Plot of genes that were significantly different (fold change ≥2 or ≤−2 and p-value adjusted ≤0.05) between the comparisons “NT vs. live” and “HK vs. live” but not “HK vs. NT.” The ten most up- and downregulated genes are in red and green, respectively. **(C)** Heatmap of genes grouping into the GO term “phagocytosis and engulfment.” The genes shown here fulfill the same criteria as in **(A)**.

Pathway analysis of the genes that were commonly downregulated (fold change ≤ −1.5 and p-value adjusted ≤ 0.05) in the two comparisons “PCLS infected with live CHA *versus* non-treated PCLS” and “PCLS infected with live CHA *versus* heat-killed CHA” revealed interesting candidates associated with cytokine production (according to the GO biological process database, **Figure 6A**). Among these 48 genes, we found several genes encoding for negative regulators of pro-inflammatory signaling such as the glycoprotein receptor CD200R1, which is also the fourth most upregulated gene (fold change: 16.9) in PCLS infected with heat-killed CHA compare to live CHA (**Figure 6B**). Interestingly, the microRNA miR-223 was significantly upregulated in PCLS infected with heat-killed compared to live CHA (**Figure 6B**). MiR-223 has been shown to initiate a signaling cascade leading to impaired IL6 production (42) as we observed it in heat-killed-CHA infected PCLS. Furthermore, we identified ten genes associated with phagocytosis and engulfment (according to the GO biological process database, **Figure 6C**) from which six genes match to the GO term “cytokine production” as well reflecting the intertwining of both processes. After analyzing the expression levels of representatives of inhibitory signaling and phagocytic uptake by quantitative RT-PCR, we decided to focus on *Cd200r1* and *Marco*. Both were higher expressed in PCLS infected with heat-killed CHA than in PCLS infected with live CHA 4 h post infection (**Supplementary Figure S5**). In summary, expression profiling revealed profound rewiring of the signaling pathways in dependency of the viability status. We could identify CD200R1 and MARCO as interesting candidates involved in the regulation of cytokine induction in PCLS.

### Robust Cytokine Induction in Viable *P. aeruginosa-*Infected PCLS Is Mediated by Bacterial Internalization

**Figure 7:**
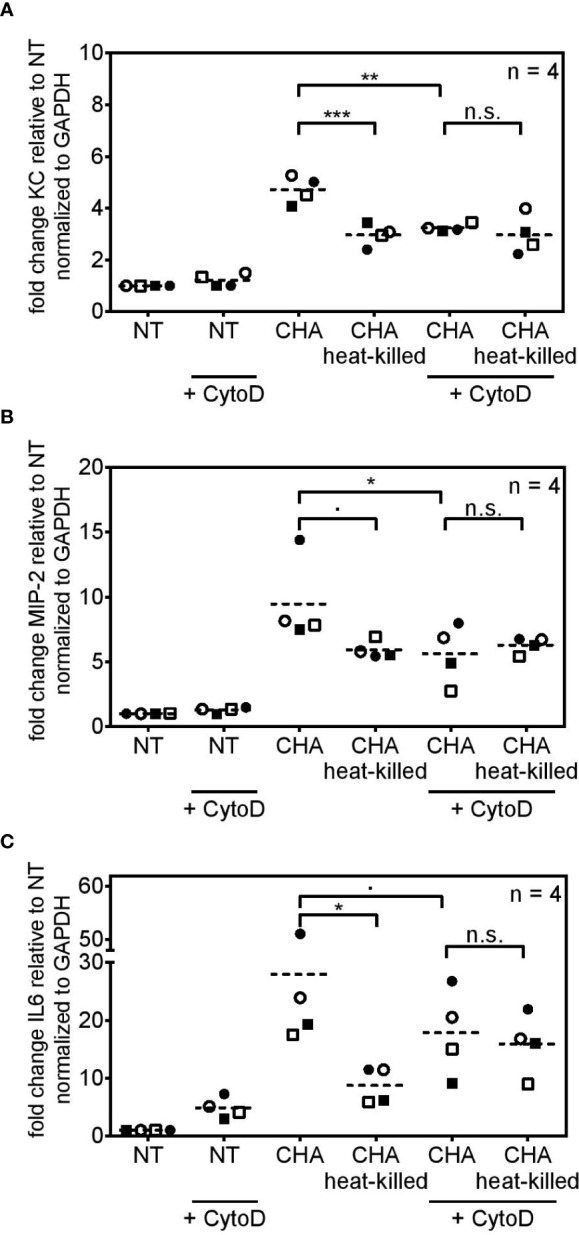
Cytokine induction in response to live CHA depends on bacterial uptake. **(A–C)** PCLS were pre-treated with cytochalasin D (+ CytoD) and subsequently infected with live CHA or heat-killed CHA or left untreated. KC **(A)**, MIP-2 **(B)**, and IL6 **(C)** expression was analyzed by quantitative real-time PCR. *Gapdh* served as housekeeping gene. Fold change was calculated by the ΔΔC_t_ method. Replicate experiments are displayed as scatter dot plot with the mean value indicated by a dashed line. Number of repetitive experiments is stated (n). n.s., not significant; ∙p=0.0619 (MIP2) or ∙p=0.0725 (IL6); *p < 0.05, **p < 0.01, ***p < 0.001.

Transcriptome analysis pointed towards a role of bacterial uptake for cytokine induction in response to an infection with CHA. Furthermore, a study by Amiel et al. reported that phagocytosis of *P. aeruginosa* depends on the flagellar swimming motility rather than on the flagellum itself (43). Hence, we set out to analyze the role of bacterial uptake for the induction of an innate immune response of PCLS towards *P. aeruginosa*. Bacterial uptake highly depends on actin-polymerization in order to engulf the pathogen (44). Hence, PCLS were treated with Cytochalasin D, a drug that inhibits actin-polymerization (45), and subsequently stimulated with live or heat-killed CHA. In the absence of bacterial uptake, PCLS were unable to differentiate between live and heat-killed CHA and cytokine induction became indistinguishable (n.s. in **Figures 7A–C**). Furthermore, inhibition of bacterial uptake significantly reduced KC (**Figures 7A**) and MIP-2 (**Figure 7B**) expression of PCLS infected with live CHA compared to live CHA-infected PCLS without Cytochalasin D treatment. Live CHA further showed a tendency to induce less IL6 expression upon Cytochalasin D-treatment, however, this reduction did not reach statistical significance (in **Figure 7C**). Inhibition of actin polymerization in PCLS did not further decrease residual cyto- and chemokine expression triggered by heat-killed CHA indicating that bacterial uptake depends on the viability of CHA. We concluded that PCLS take up live *P. aeruginosa* und subsequently trigger cytokine expression. In contrast, dead *P. aeruginosa* is not internalized but may trigger moderate cytokine release *via* TLR signaling. We concluded that internalization of live *P. aeruginosa via* phagocytosis into PCLS is required for potent cytokine induction.

### CD200R1 and MARCO Are Involved in the Uptake of Live *P. aeruginosa* in PCLS

**Figure 8:**
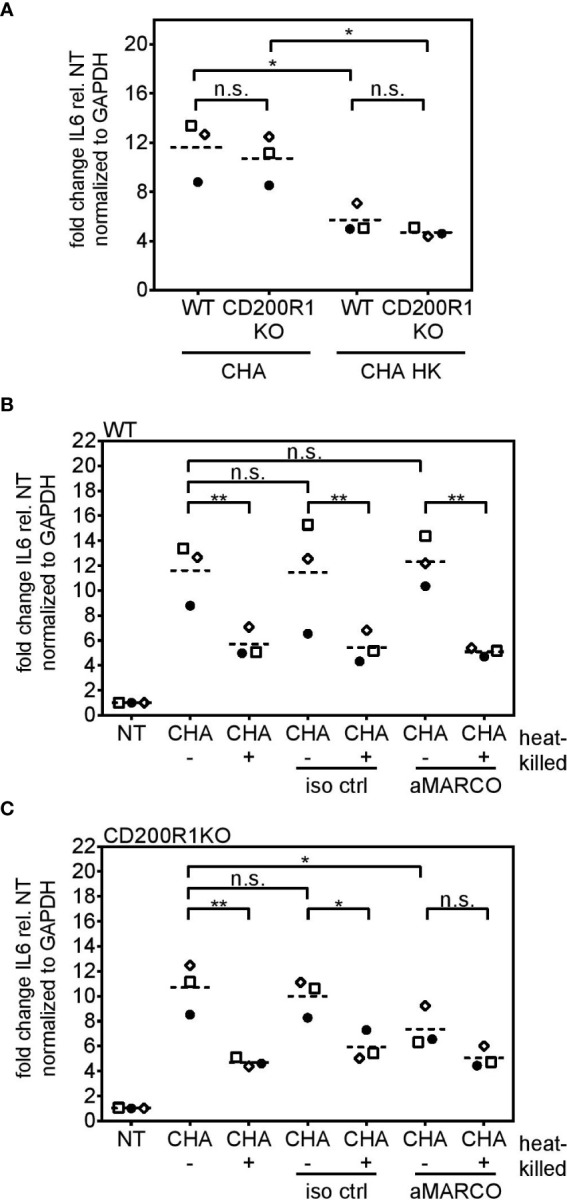
*MARCO* and *CD200R1* are redundantly involved in the uptake of CHA in PCLS. **(A)** PCLS were generated from C57BL/6J (WT) and CD200R1 KO mice and infected with live or heat-killed (HK) CHA. WT **(B)** and CD200R1KO **(C)** PCLS were pre-treated with an antibody against the phagocytic receptor MARCO (aMARCO) or an isotype control (iso ctrl) before infection with live (−) or heat-killed (+) CHA. IL6 expression was analyzed by quantitative real-time PCR. Values were normalized to *Gapdh*. Fold change was calculated by the ΔΔC_t_ method. **(A–C)** Replicate experiments are displayed as scatter dot plot with the mean value indicated by a dashed line. Number of repetitive experiments is n = 3. n.s., not significant; *p < 0.05, **p < 0.01.

*Cd200r1* encodes a cell surface glycoprotein that downregulates pro-inflammatory signaling (29, 46). Additionally, Chen et al. showed that CD200, the ligand of CD200R1, triggers phagocytosis (47). WT and CD200R1 PCLS showed a similar IL6 induction upon infection with live or heat-killed CHA (**Figure 8A**). Besides CD200R1, we were interested in the role of the non-opsonic phagocytic receptor MARCO which was highlighted by the functional enrichment analysis (GO term “phagocytosis and engulfment”). However, antibody-mediated inhibition of MARCO (**Figure 8B**) did not affect live/dead discrimination in PCLS infected with live or heat-killed CHA. Yet, infection of CD200R1 KO PCLS with live CHA and simultaneous inhibition of MARCO significantly decreased IL6 expression compared to live CHA-infected CD200R1 KO PCLS with functional MARCO signaling (**Figure 8C**). Furthermore, in the absence of functional CD200R1 and MARCO, PCLS do not discriminate between live and heat-killed CHA anymore (**Figure 8C**). However, the effect was most pronounced for IL6 expression and the absence of both receptors had a rather weak impact on KC and MIP-2 expression arguing for a cytokine-dependency (**Supplementary Figure S6**). These results imply a redundant involvement of both receptors, CD200R1 and MARCO, in the internalization of *P. aeruginosa* and subsequently IL6 induction.

## Discussion

Breathing allows access of live and dead airborne microbes to the lung illustrating the risk of constant inflammation and detrimental immune reactions (3, 4). Accordingly, it is of utmost importance for the pulmonary microenvironment to evaluate the level of threat arising from inhaled encounters. PCLS, which are small sections of the lung, represent a powerful tool to specifically understand the innate immune reactions in the lung as an interplay between different pulmonary cells unbiased of parallel ongoing whole-body processes (48, 49). In this context, we employed murine PCLS to investigate live/dead discrimination of *P. aeruginosa*. In the presented study, we provide evidence for four major findings: (I) PCLS discriminate between live and dead *P. aeruginosa* infection. (II) Cytokine induction in PCLS depends on the flagellar-filament protein fliC and the T3SS, with special emphasis on the needle protein pscF. (III) PCLS specifically internalize live *P. aeruginosa*. (IV) MARCO and CD200R1 are redundantly involved in the internalization of live *P. aeruginosa* and IL6 induction.

In this study, we show that PCLS discriminate between an encounter with live or dead *P. aeruginosa*. A previous landmark study by Sander et al. (5) introduced the concept of live/dead discrimination by showing that production of IFNβ and IL1β are strongly reduced or even absent in murine bone marrow-derived macrophages in response to heat-killed compared to live *E. coli*, respectively. They showed that *E. coli*-derived mRNA serves as a vita-PAMP specifically associated with live bacteria, thus signaling microbial danger and inducing a more complex immune response in infected cells (5). In case of *P. aeruginosa*, live/dead discrimination so far was elusive. Several studies demonstrate that the innate immune response towards *P. aeruginosa* is mainly transmitted *via* the classical PAMPs LPS and/or flagellin and their receptors TLR4 or TLR5, respectively (9, 10, 11, 28). Yet, we now show in this study that PCLS specifically sense the viability of *P. aeruginosa* and initiate a complex and profound rewiring of their transcriptome in response to live but not heat-killed *P. aeruginosa*. We show that robust early cytokine expression such as KC, MIP-2, and IL6 is initiated in response to live *P. aeruginosa* but is significantly impaired once the bacterium is heat-killed, emphasizing an involvement of vita-PAMPs in the recognition process of *P. aeruginosa*. We specifically monitored KC and MIP-2 levels since both chemokines are involved in mounting an early innate immune response by recruiting neutrophils (50, 51) and further analyzed IL6 levels since this cytokine is known to extent but also to regulate pro-inflammatory actions (52, 53). The observation that the viability status of the pathogen reflects the capacity to induce a distinct cytokine profile was also observed by Cruciani et al. (54) for another pathogen: They showed that live *S. aureus*-infected DCs release IL1β, IL12, TNFα, IL23, IL6, and IL8, whereas the release of these cytokines was significantly reduced upon heat-killing *S. aureus* resulting in a differential Th1/Th17 response. In summary, a major finding of our study is that PCLS scale early cyto- and chemokine induction to the viability status of *P. aeruginosa* with viable but not dead bacterial cells acting as inducers of pro-inflammatory gene expression.

*In vitro* data of Raoust et al. (11) suggest that the release of KC and IL6 by murine alveolar macrophages and epithelial cells in response to *P. aeruginosa* is mediated by sensing flagellin *via* TLR5 and LPS via TLR2/4. *In vivo* studies examining BAL fluid and lung homogenates of infected mice further support the assumption that release of KC, IL6, and MIP-2 relies on host cell-expression of TLR2 and/or TLR4 and flagellin-expressing *P. aeruginosa*, however, each cytokine shows a predominant but still redundant dependency (9, 10). In contrast to these studies, we specifically compared the recognition of live and heat-killed *P. aeruginosa* mutants by PCLS and hence, our findings of viability-dependent recognition of *P. aeruginosa* further expand earlier studies on the structural requirements of *P. aeruginosa* for the induction of an innate immune response. We were able to attribute the significant gap in the expression of classical NFκB-dependent cytokines between PCLS infected with live and heat-killed *P. aeruginosa* mutants to a redundant recognition of flagellin and the T3SS, with the latter playing a minor role. In contrast to the findings of Raoust et al. (11), we exclude the classical role of flagellin as ligand of the surface-localized TLR5 by performing infection studies with TLR5 KO PCLS. This discrepancy may be explained with the higher cellular complexity of PCLS compared to studies with a single isolated cell type. The *in vivo* studies by Ramphal et al. do not exclude a role of TLR5 as we did, but they show that *P. aeruginosa* expressing flagellin, unable to bind TLR5, is less toxic than the *P. aeruginosa* strain lacking flagellin expression completely, hinting towards a less profound role of the flagellin-TLR5 interaction in a more complex system (10). Finally, Morris et al. (55) investigated whole lung homogenates of *P. aeruginosa*-infected wildtype and TLR5 KO mice and showed that TLR5 is negligible for KC and IL6 production at 4 h post infection, an observation that is in line with our findings. Hence, we concluded that flagellin does not play a role as simple PAMP for differentiating live and dead *P. aeruginosa* but serves as a vita-PAMP in this context.

Flagellar swimming motility is an energy-consuming feature (41) that uniquely characterizes live bacteria and could explain the significant higher cytokine induction in response to live compared to heat-killed *P. aeruginosa*, even though both carry flagellin as part of their flagellum on their surface. A previous study by Amiel et al. reported that the flagellar swimming motility rather than expression of the flagellum itself is a prerequisite for phagocytic internalization of *P. aeruginosa* (43). We, in our study, demonstrate that under conditions of blocked bacterial uptake, live and heat-killed *P. aeruginosa* exhibit a similar capacity to induce cyto- and chemokines, indicating that internalization boosts the early innate immune response towards viable *P. aeruginosa*. Interestingly, blocking of bacterial uptake did not further decrease the cytokine response towards heat-killed *P. aeruginosa* emphasizing that viability is a prerequisite for bacterial uptake. In accordance with our findings, Speert et al. (56) reported impaired phagocytic uptake of heat-killed and UV-irradiated *P. aeruginosa* in polymorphonuclear leukocytes and monocyte-derived macrophages. We therefore suggest that extracellular pathways leading to the activation of the innate immune response towards *P. aeruginosa* are complemented by intracellular signaling events upon bacterial uptake culminating in an enhanced cyto- and chemokine response. Previous work showed that *P*. a*eruginosa* is able to escape the phagosome and induce macrophage disruption (57), illustrating the importance of understanding intracellular detection pathways that contribute to the amplification of the very early innate immune response taking place right before internalized viable *P. aeruginosa* can unfold its infectious capacities by secreting T3SS-dependent toxins. To date, it is not clear which cell types within PCLS are involved in the uptake of *P. aeruginosa* but we expect an important role of airway epithelial cells as non-professional phagocytic cells. Once internalized, cytokine activation may occur *via* sensing of unmethylated CpG-motifs of *P. aeruginosa* DNA by endosomal TLR9 (58, 59) or by sensing diaminopimelate-containing muropeptides of the bacterial peptidoglycan *via* cytosolic Nod1 (15). Additionally, intracellular detection of flagellin or T3SS components activates the IPAF (NLRC4) inflammasome finally leading to IL1β formation (60, 61). Live CHA indeed triggered a robust release of the IL1α and IL1β in PCLS whereas heat-killed CHA did not, highlighting the importance of *Pseudomonas* viability and/or internalization for the early innate immune response. Ultimately, cytotoxic secondary bacterial actions upon host cell contact and internalization are a main reason for the early innate immune response to rely on the recognition of vita-PAMPs besides sensing of the classical PAMPs.

Besides the role of flagellin for cytokine induction in response to live *P. aeruginosa*, we could show that the T3SS and specifically the needle protein pscF contribute to KC expression in PCLS infected with live *P. aeruginosa*. Since the expression and assembly of the T3SS is initiated as soon as bacteria sense host cell contact (40), T3SS-associated structural elements such as the needle protein are characteristic features of live bacteria explaining the importance for live/dead discrimination. Interestingly, we observed that heat-killed CHA fails to induce IL1-cytokine secretion and we attribute that failure to the absence of vita-PAMPs such as the flagellum und the T3SS. Indeed, Descamps et al. showed that IL1β release is impaired in alveolar macrophages infected with PAKΔpscF or PAKΔflic (62). Our data are further in agreement with a study by Jessen et al. (63) showing that the purified T3SS needle proteins YscF of *Yersinia pestis*, PrgI and SsaG of *Salmonella enterica* and MxiH of *Shigella* induce MyD88-dependent NFκB-activation and cytokine release (IL6, IL8, TNFα) in THP1 cells. They further propose that the needle proteins act vi*a* TLR2 and TLR4 to activate NFκB- and AP1-dependent gene expression. Based on our results and the fact that YscF of *Y. pestis* as well as pscF of *P. aeruginosa* belong to the Ysc-type of injectisomes (64), we consider the hypothesis that pscF acts as an immunogenic feature triggering NFκB-dependent gene expression as a reasonable assumption. Previous studies (10, 11) assume that the involvement of TLR2/4 in the recognition of *P. aeruginosa* can be attributed to LPS, which is certainly true, but the receptor activity may be further complemented by the engagement with T3SS-associated structural proteins such as the needle protein pscF. Hence, our results agree with previous findings, provide additional insights into the recognition process and outline live/dead discrimination of *P. aeruginosa*.

Up to now, little was known about the host cell receptors that are required for initiating *Pseudomonas* uptake. In the pulmonary microenvironment, non-opsonic phagocytosis likely represents the predominant type of phagocytosis since mediators of the complement system and antibody levels are rather low (56). To date, several phagocytic receptors have been reported to mediate the uptake of *P. aeruginosa*, however, engagement with the one or the other depends on the pseudomonas strain and its surface moieties (65, 66). In our study, we provide evidence that MARCO and CD200R1 are involved in the bacterial uptake of viable *P. aeruginosa* in PCLS, potentially triggered by vita-PAMPs, and IL6 induction subsequently. Of note, either receptor was sufficient to induce IL6 expression in *P. aeruginosa*-infected PCLS, arguing for a redundancy between MARCO and CD200R1. A previous study by Domingo-Gonzalez et al. (67) showed that phagocytic uptake of *P. aeruginosa* is strongly reduced upon treatment of alveolar macrophages with soluble MARCO. Furthermore, Descamps et al. (62) suggest that TLR5 plays a non-redundant role in the phagocytic uptake of *P. aeruginosa* in alveolar macrophages. We explain these discrepancies with the higher cellular complexity of PCLS in which individual and cell-type specific dependencies may be masked. The involvement of CD200R1 in the uptake of live *P. aeruginosa* was surprising knowing its well-established function as inhibitor of pro-inflammatory signaling (29, 46, 68). Our first assumption that heat-killed *P. aeruginosa* is not able to overcome the limiting actions of CD200R1 reflecting in a diminished pro-inflammatory cytokine profile did not prove as being true. Our finding that CD200R1 is involved in the uptake of live *P. aeruginosa* is supported by a recent study showing that the uptake of CD200-coated PLGA, a biomaterial, is enhanced by CD200R1-expressing macrophages compared to non-coated PLGA (47). Accordingly, activation of CD200R1 by its ligand CD200 may activate a signaling cascade culminating in improved bacterial uptake and in the induction of IL6. The seemingly contradictory finding of MARCO and CD200R1 downregulation in PCLS infected with live *P. aeruginosa* compared to heat-killed *P. aeruginosa versus* the potential role in mediating bacterial uptake of live *P. aeruginosa* may be explained be a feedback mechanism. We assume that both receptors mediate the uptake of *P. aeruginosa* in the very beginning of an infection and upon internalization, both are downregulated either by the host cell to prevent an overshooting immune reaction or by the pathogen to evade further immune detection and promote survival. Downstream signaling events such as those elicited by the T3SS effector toxin ExoS may lead to phagosomal escape and inhibition of phagocytic uptake at later time points (57, 69). To further elucidate the involvement of MARCO and CD200R1 in the bacterial uptake of *P. aeruginosa*, a confocal microscopic approach might be helpful in which *P. aeruginosa* and receptors are fluorescently labeled to visualize a potential co-localization. However, since PCLS are tissue sections that float in their culture medium, experience is needed to implement such an experiment.

In conclusion, the results of our study demonstrate that PCLS initiate a more intense early cyto- and chemokine response towards live than heat-killed *P. aeruginosa* by specifically internalizing viable bacteria characterized by a functional flagellum and a T3SS. We further show, that bacterial uptake is partially and redundantly mediated by MARCO and CD200R1. Future studies may focus specifically on dissecting the interaction between viable *P. aeruginosa* and the host cell receptors MARCO and CD200R1 and on defining intracellular detection pathways that account for the lack of pro-inflammatory gene induction in response to dead *P. aeruginosa*. Generally, for understanding the early innate immune response towards *P. aeruginosa* as an interplay between pulmonary cells, single cell sequencing of infected PCLS would provide further insights.

## Data Availability Statement

The datasets presented in this study can be found in online repositories. The names of the repository/repositories and accession number(s) can be found below: https://www.ebi.ac.uk/ena, SAMEA7231371-7231379.

## Ethics Statement

The animal study was reviewed and approved by the local authorities (animal welfare Officer, University Hospital Heidelberg) according to the local animal welfare rules.

## Author Contributions

UK performed and analyzed experiments. SB conducted the bioinformatic analysis of the transcriptome data. BY performed the functional profiling of the transcriptome data. Transcriptome-related graphics were generated by SB, BY and UK. UK and SB performed the statistical analysis. TP performed histological analysis of H&E stained PCLS. AS provided the CD200R1 KO mice. SB and AD designed, supported, and supervised the study. UK, SB, and AD prepared the manuscript. All authors contributed to the article and approved the submitted version.

## Funding

This work was supported by a grant of the German Research Foundation to AD (DFG Da592/6-1).

## Conflict of Interest

The authors declare that the research was conducted in the absence of any commercial or financial relationships that could be construed as a potential conflict of interest.
