# Supplementary material for: Early Cytokine Induction Upon Pseudomonas aeruginosa Infection in Murine Precision Cut Lung Slices Depends on Sensing of Bacterial Viability
Source: Front Immunol. 2020 Oct 30;11:598636. doi: 10.3389/fimmu.2020.598636 (PMC7673395; doi:10.3389/fimmu.2020.598636)
Supplement: Supplementary file 1 [file DataSheet_1.pdf]

Supplementary Material

1 Supplementary Figures and Tables

1.1 Supplementary Figures

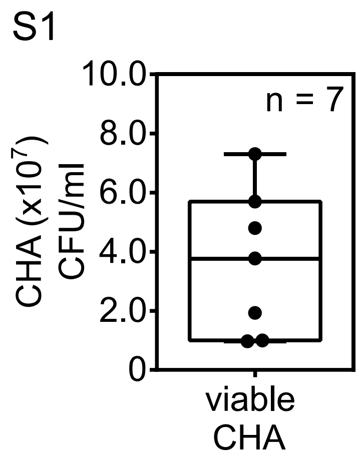

Figure S1: PCLS were infected with approximately 3.64 x 10^5 viable CFU.

For infections, a CHA suspension was adjusted to a McFarland value of 0.5 and 10 µl thereof were added to PCLS. The number of viable bacteria in the CHA suspension was assessed with the QUANTOM™ Viable Cell Staining Kit. Values are represented in a box plot with whiskers ranging from the minimal to the maximal data point. Based on seven independent experiments.

S2

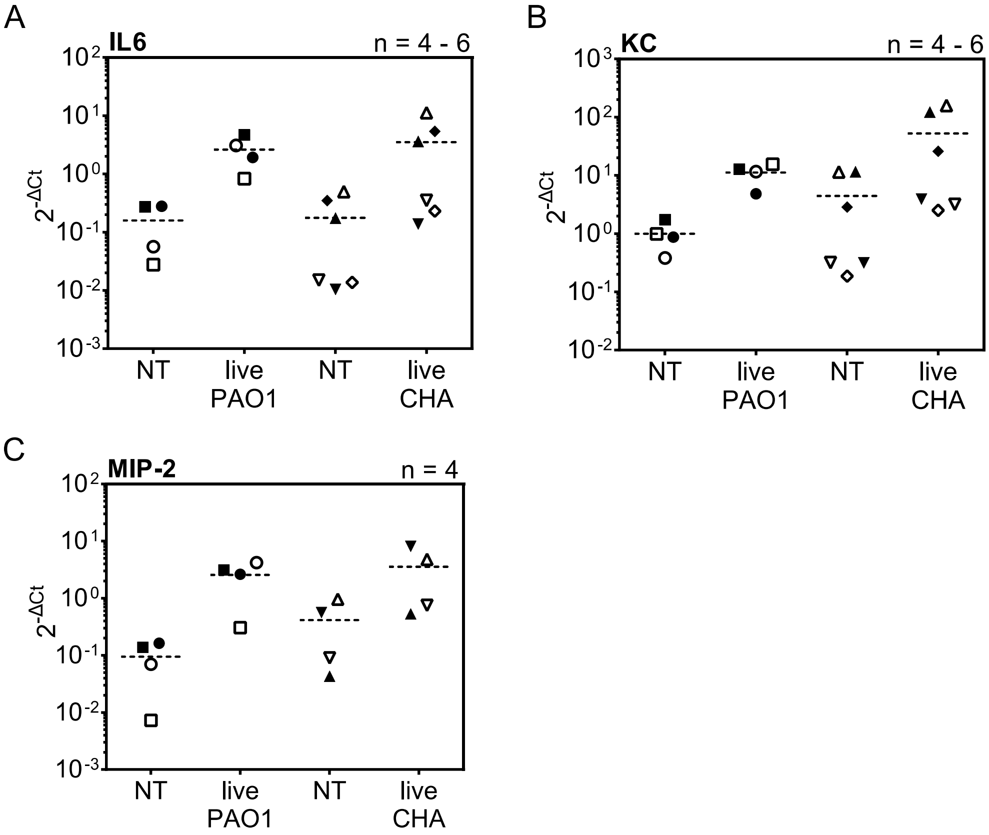

Figure S2: PAO1 and CHA have a similar IL6- and KC inductive capacity.

PCLS were either left untreated (NT) or infected with live PAO1 or live CHA for four hours. IL6 (A), KC (B) and MIP-2 (C) expression were analyzed by real-time PCR. The C_t value of the housekeeping gene was subtracted from the C_t value of the gene of interest. To allow comparisons between the absolute IL6 and KC inductive capacities of both strains, the $2^{-\Delta Ct}$ value is plotted. Data are represented in scatter dot plots with the mean values indicated by a dashed line. Number of repetitive experiments is stated (n).

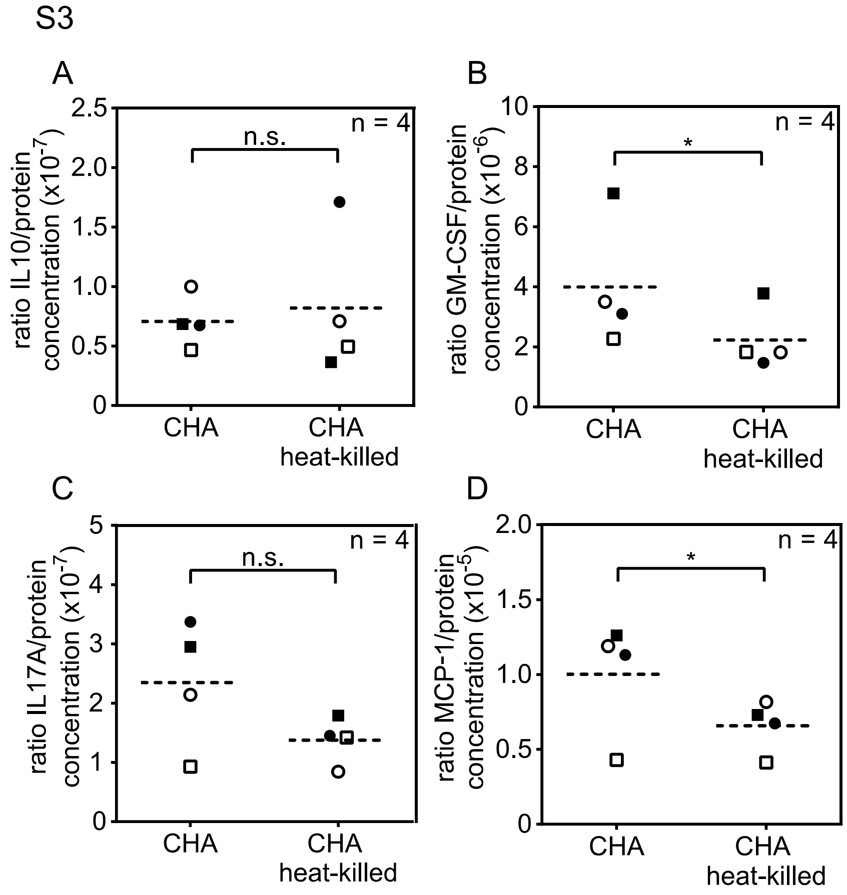

Figure S3: Viability dependent regulation of protein secretion.

(A-D) Conditioned supernatant of PCLS infected with live or heat-killed CHA was analyzed with the LEGENDplex Mouse Inflammation Panel six hours post infection. IL10 (A), GM-CSF (B), IL17A (C) and MCP-1 (D) concentrations were normalized to the total protein concentration to correct for PCLS-tissue size differences. (A-D) Data are represented as scatter dot plots with the mean value indicated by a dashed line. Number of repetitive experiments is stated (n). n.s. = not significant, *$p < 0.05$

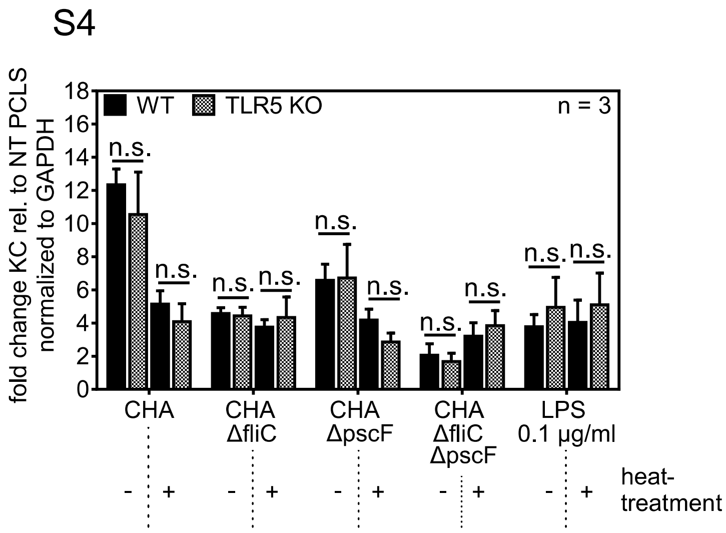

Figure S4: Live/dead discrimination is independent of TLR5-mediated sensing of flagellin.

PCLS were generated from wildtype and TLR5 KO mice and stimulated with live or heat-killed CHA, CHA ΔfliC, CHA ΔpscF or CHA ΔfliCΔpscF. LPS stimulation was used as control for TLR5-independent stimulation. KC expression was analyzed four hours post infection on RNA level by qRT-PCR. *Gapdh* was used as housekeeping gene. Fold change was calculated by the $\Delta\Delta C_t$ method. Values represent mean data (± SEM) of three independent experiments.

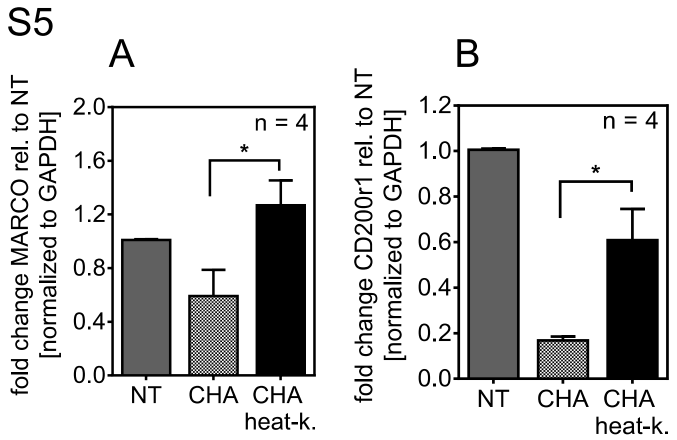

Figure S5: *Marco* and *CD200r1* are significantly different expressed in PCLS infected with live and heat-killed CHA.

PCLS were infected with live or heat-killed (heat-k.) CHA or left untreated (NT) and expression levels of *Marco* (I) and *Cd200r1* (II) were analyzed four hours post infection. *Gapdh* served as housekeeping gene. Fold change was calculated by the $\Delta\Delta C_t$ method. Bar blots represent mean data (± SEM) of four independent experiments. $*p < 0.05$

S6

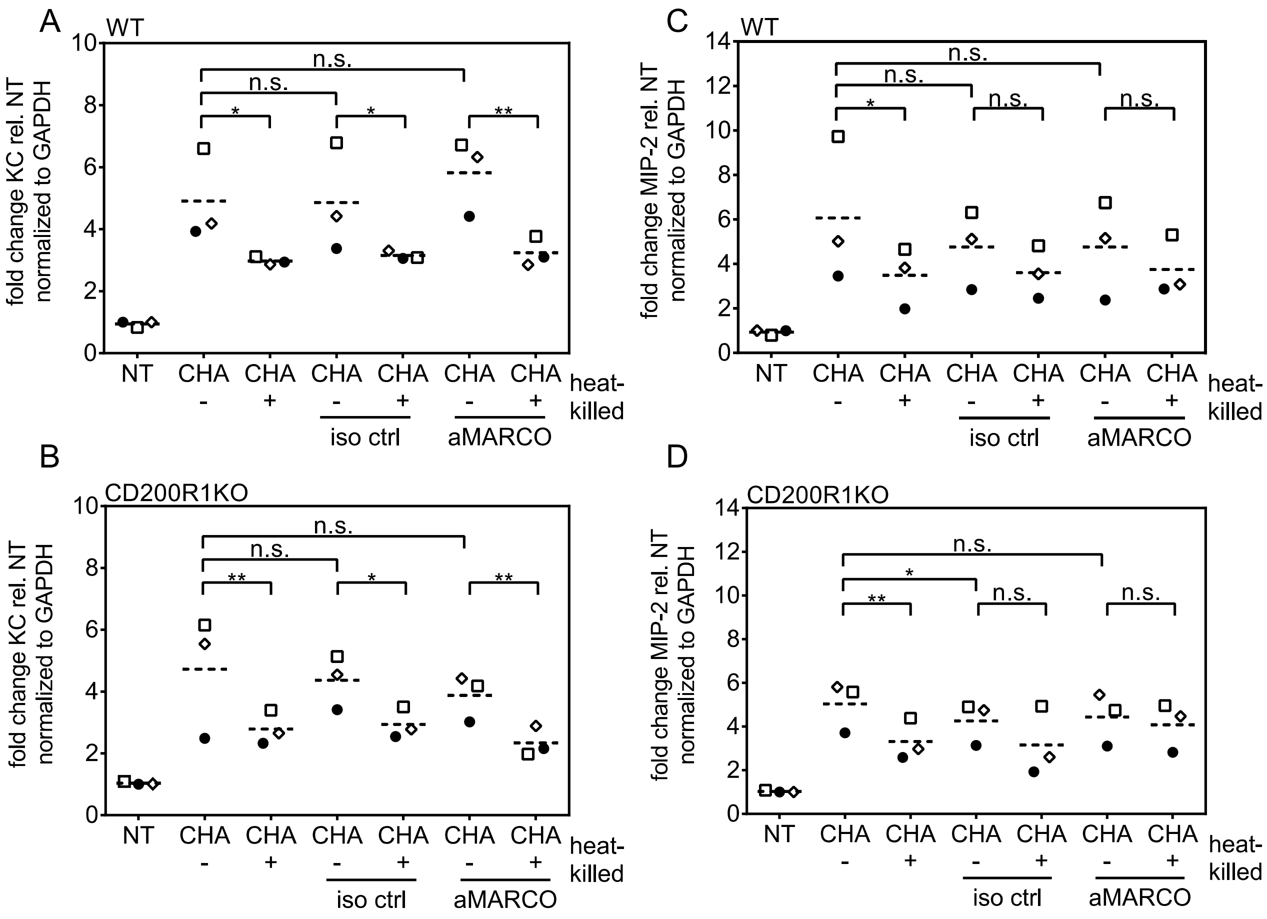

Figure S6: Redundant role of MARCO and CD200R1 in the uptake of live CHA

(A-D) PCLS were generated from WT and CD200R1KO mice and either left naïve or pre-treated with an isotype control (iso ctrl) or an anti-MARCO antibody (aMARCO) before further infection. PCLS were stimulated with live (-) or heat-killed (+) CHA or left untreated (NT). Expression levels of KC (A, B) and MIP-2 (C, D) were analyzed four hours post infection. *Gapdh* served as housekeeping gene. Fold change was calculated by the $\Delta\Delta C_t$ method. Scatter dot plots represent biological replicates (n=3) with the mean value indicated by a dashed line. n.s. = not significant, *p< 0.05; **p < 0.01

1.2 Supplementary Tables

Table T1*:* LIVE *versus* HK ten most downregulated pathways.

ID	Description	Gene ratio	qvalue	Genes
GO:0006952	defense response	99/362	6,13E-11	Nlrp1b/Cd200r1/Abcd2/Cd300lf/Tlr8/Arg1/Trim29/Myo1f/Cx3cr1/B430306N03Rik/Trem2/Tlr13/Havcr2/Ccr1/Fcgr1/Itgax/Clec5a/Rab7b/Clec4a2/C3ar1/Spn/Cybb/Cmklr1/Slamf8/Alox5/Cd200r4/Chil3/Itgb2/Lilra5/Marco/Ccl9/Mpeg1/Tril/Lpl/Sh2d1b1/Mgll/Siglece/Alox5ap/Cd84/Fpr1/Pycard/Csf1r/Wnt5a/Naip6/Fpr3/Naip2/Aoah/Card9/Fcgr3/Cd300a/Ctss/Naip5/Tlr7/Tyrobp/Fgr/C1qc/Tarm1/Cd68/Lipa/Bst1/Fcer1g/Acp5/Mrc1/A530064D06Rik/Fcgr2b/Slc11a1/Pla2g7/Pik3cg/Clec4n/C5ar1/Pf4/Ltb4r1/C1qa/Nlrc4/Sirpa/Chil4/Pik3ap1/Hck/Vav1/Gng7/Olr1/Ly9/Clec4d/Cyp26b1/C1qb/Il18/Cfp/Tnfaip8l2/Clec4e/Syk/Zfp580/Aif1/Pik3r6/Chst2/Il1a/Mapk14/Igf1/Cotl1/Ppbp
GO:0006955	immune response	81/362	3,92E-06	Nlrp1b/Cd300lf/Nfam1/Tlr8/Arg1/Trim29/Myo1f/Cx3cr1/B430306N03Rik/Trem2/Tlr13/Havcr2/Ccr1/Fcgr1/Clec5a/Rab7b/Clec4a2/C3ar1/Spn/Cybb/Cmklr1/Slamf8/Itgb2/Adgre1/Marco/Ccl9/P2ry14/Tril/Sh2d1b1/Cd84/Fpr1/Pycard/Csf1r/Wnt5a/Naip6/Fpr3/Naip2/Card9/March1/Fcgr3/Pirb/Cd300a/Naip5/Tlr7/Tyrobp/Fgr/C1qc/Tarm1/Cd68/Fcer1g/Mrc1/A530064D06Rik/Lcp1/Clec12a/Fcgr2b/Slc11a1/Clec4n/C5ar1/Pf4/C1qa/Lcp2/Nlrc4/Sirpa/Pik3ap1/Hck/Vav1/Ly9/Clec4d/Card11/C1qb/Il18/Cfp/Tnfaip8l2/Clec4e/Syk/Lfng/Pik3r6/Il1a/Fyb/Mapk14/Ppbp
GO:0002682	regulation of immune system process	77/362	2,39E-04	Cd200r1/Cd300lb/Cd300lf/Nfam1/Tlr8/F7/Arg1/Myo1f/B430306N03Rik/Trem2/Tlr13/Havcr2/Ccr1/Fcgr1/Rab7b/C3ar1/Spn/Cmklr1/Slamf8/Itgb2/Tril/Sh2d1b1/Cd84/Fpr1/Pycard/Csf1r/Wnt5a/Fpr3/Card9/Fcgr3/Mitf/Cd300a/Mmp28/Tlr7/Tyrobp/Fgr/C1qc/Tarm1/Cd68/Bst1/Hoxa7/Fcer1g/A530064D06Rik/Gpnmb/Clec12a/Fcgr2b/Slc11a1/Pla2g7/Spi1/C5ar1/Pf4/C1qa/Pilrb1/Lcp2/Nlrc4/Sirpa/Pik3ap1/Csf3r/Vav1/Clec4d/Card11/Cyp26b1/C1qb/Il18/Cfp/Tnfaip8l2/Clec4e/Syk/Zfp580/Aif1/Mafb/Pik3r6/Il1a/Fyb/Mapk14/Igf1/Slfn1
GO:0006954	inflammatory response	71/362	2,97E-10	Nlrp1b/Cd200r1/Abcd2/Tlr8/Cx3cr1/Trem2/Tlr13/Havcr2/Ccr1/Fcgr1/C3ar1/Spn/Cybb/Cmklr1/Slamf8/Alox5/Cd200r4/Chil3/Itgb2/Lilra5/Ccl9/Tril/Lpl/Mgll/Siglece/Alox5ap/Fpr1/Pycard/Csf1r/Wnt5a/Naip6/Fpr3/Naip2/Aoah/Fcgr3/Cd300a/Ctss/Naip5/Tlr7/Tyrobp/Tarm1/Cd68/Lipa/Bst1/Fcer1g/Acp5/Fcgr2b/Slc11a1/Pla2g7/Pik3cg/C5ar1/Pf4/Ltb4r1/C1qa/Nlrc4/Sirpa/Chil4/Pik3ap1/Hck/Olr1/Cyp26b1/Il18/Tnfaip8l2/Syk/Zfp580/Aif1/Chst2/Il1a/Mapk14/Igf1/Ppbp
GO:0002684	positive regulation of immune system process	58/362	1,49E-03	Cd300lb/Cd300lf/Nfam1/Tlr8/F7/Arg1/Trem2/Tlr13/Havcr2/Ccr1/Fcgr1/Rab7b/C3ar1/Spn/Cmklr1/Itgb2/Tril/Sh2d1b1/Cd84/Fpr1/Pycard/Csf1r/Wnt5a/Fpr3/Card9/Fcgr3/Cd300a/Tlr7/Tyrobp/Fgr/C1qc/Bst1/Fcer1g/Fcgr2b/Slc11a1/Pla2g7/C5ar1/Pf4/C1qa/Lcp2/Nlrc4/Sirpa/Pik3ap1/Vav1/Clec4d/Card11/C1qb/Il18/Cfp/Clec4e/Syk/Zfp580/Aif1/Pik3r6/Il1a/Fyb/Mapk14/Igf1
GO:0001816	cytokine production	56/362	5,59E-04	Adam33/Nlrp1b/Cd200r1/Abcd2/Nfam1/Tlr8/Arg1/Cx3cr1/Trem2/Havcr2/Clec5a/Rab7b/Clec4a2/C3ar1/Spn/Cybb/Cmklr1/Lilra5/Tril/Lpl/Sh2d1b1/Cd84/Pycard/Csf1r/Wnt5a/Card9/Fcgr3/Cd300ld/Naip5/Tlr7/Tyrobp/Fgr/Tarm1/Lipa/Fcer1g/Acp5/Gpnmb/Fcgr2b/Slc11a1/Clec4n/C5ar1/Pf4/Lcp2/Maf/Nlrc4/Sirpa/Ly9/Card11/Il18/Clec4e/Syk/Zfp580/Aif1/Il1a/Mapk14/Igf1
GO:0045087	innate immune response	55/362	1,82E-06	Nlrp1b/Cd300lf/Tlr8/Arg1/Trim29/Myo1f/Cx3cr1/B430306N03Rik/Trem2/Tlr13/Havcr2/Ccr1/Fcgr1/Clec5a/Rab7b/Clec4a2/Cybb/Slamf8/Marco/Ccl9/Tril/Sh2d1b1/Cd84/Pycard/Csf1r/Wnt5a/Naip6/Naip2/Card9/Cd300a/Naip5/Tlr7/Fgr/C1qc/Tarm1/Fcer1g/Mrc1/A530064D06Rik/Slc11a1/Clec4n/C1qa/Nlrc4/Sirpa/Pik3ap1/Hck/Vav1/Ly9/Clec4d/C1qb/Il18/Cfp/Tnfaip8l2/Clec4e/Syk/Pik3r6
GO:0001817	regulation of cytokine production	52/362	1,49E-03	Adam33/Nlrp1b/Cd200r1/Abcd2/Nfam1/Tlr8/Arg1/Cx3cr1/Trem2/Havcr2/Clec5a/Rab7b/Clec4a2/C3ar1/Spn/Cybb/Cmklr1/Lilra5/Tril/Lpl/Sh2d1b1/Cd84/Pycard/Csf1r/Wnt5a/Card9/Fcgr3/Cd300ld/Naip5/Tlr7/Tyrobp/Fgr/Tarm1/Fcer1g/Acp5/Gpnmb/Fcgr2b/Slc11a1/Clec4n/C5ar1/Pf4/Sirpa/Ly9/Card11/Il18/Clec4e/Syk/Zfp580/Aif1/Il1a/Mapk14/Igf1
GO:0031347	regulation of defense response	51/362	4,15E-05	Cd200r1/Abcd2/Cd300lf/Tlr8/Arg1/Myo1f/Cx3cr1/B430306N03Rik/Trem2/Tlr13/Havcr2/Ccr1/Fcgr1/Rab7b/Spn/Slamf8/Cd200r4/Lilra5/Tril/Lpl/Sh2d1b1/Mgll/Siglece/Alox5ap/Pycard/Wnt5a/Aoah/Card9/Fcgr3/Cd300a/Ctss/Tlr7/Fgr/Tarm1/Bst1/Fcer1g/Acp5/A530064D06Rik/Fcgr2b/Pik3cg/C5ar1/C1qa/Nlrc4/Sirpa/Pik3ap1/Vav1/Tnfaip8l2/Clec4e/Pik3r6/Mapk14/Igf1
GO:0050776	regulation of immune response	50/362	4,08E-04	Cd300lf/Nfam1/Tlr8/Arg1/Myo1f/B430306N03Rik/Tlr13/Havcr2/Ccr1/Fcgr1/Rab7b/C3ar1/Spn/Cmklr1/Slamf8/Itgb2/Tril/Sh2d1b1/Cd84/Fpr1/Pycard/Wnt5a/Fpr3/Card9/Fcgr3/Cd300a/Tlr7/Fgr/C1qc/Fcer1g/A530064D06Rik/Clec12a/Fcgr2b/Slc11a1/C5ar1/C1qa/Lcp2/Nlrc4/Pik3ap1/Vav1/Clec4d/Card11/C1qb/Il18/Cfp/Clec4e/Syk/Pik3r6/Fyb/Mapk14

GO, gene ontology biological process terms. Genes showing differential expression (FC<-1.5, adjusted p-value ≤ 0.05) were used as input for the analysis.

Table T2: LIVE vs HK ten most upregulated pathways.

ID	Description	Gene ratio	qvalue	Genes
GO:0009605	response to external stimulus	91/296	2,73E-07	Nppa/Ifng/Nr4a3/Il17a/Tslp/Il17f/Il6/Nr4a2/Nr4a1/Ptgs2/Serpine1/Noct/Cxcl1/Ccrl2/Il23a/Ripk2/Duox2/Ptx3/Thbs1/Penk/Ier3/Cxcl2/Tnfsf8/Tnfaip3/Pgf/Vcam1/Tnfsf11/Atf3/Gdf15/Tnfaip6/C2cd4b/B2m/Sik1/Fos/Nfkbiz/Dusp1/Adamts4/Adamts9/Bcl2l11/Sox9/Trpv4/Vgf/Pde4b/Cxcl11/Nod2/Ccl11/Selp/Slc25a25/Tnip1/Serpina3f/Bmp6/Ryr2/Cflar/Prkcg/Grem1/Inhbb/Tnnt2/Zfp36/Klf4/Bdnf/Bcl3/Nfkbia/Bhlhe40/Ttn/Nfkb1/Cdk5r1/Arid5a/Ddit4/Plau/Slc2a1/Nrg1/Cxcl10/Pdgfa/Cx3cl1/Nos2/Thbd/Hbegf/Sema4c/Junb/Ccl2/Gbp5/Depdc5/Cd40/Ptpn2/Bmp2/Zswim6/Pdgfb/Chd7/Il2ra/Cxcl9/Fosl1
GO:0048584	positive regulation of response to stimulus	90/296	3,69E-07	Nppa/Ifng/Il3/Nr4a3/Il17a/Fgf23/Csf2/Tslp/Il5/Il17f/Cacng4/Il6/Akap12/Tnfsf15/Lif/Il11/Ptgs2/Serpine1/Cxcl1/Ereg/Il23a/Ripk2/Duox2/Thbs1/Csf3/Foxc2/Penk/Tnfaip3/Pgf/Tnfsf11/Atf3/Gdf15/C2cd4b/B2m/Ctla4/Nfkbiz/Bcl2l11/Sox9/Trpv4/Dll1/Pde4b/Nod2/Ccl11/Selp/Tnip1/Rell1/Bmp6/Ppp1r15a/Mmp3/Cflar/Prkcg/Grem1/Inhbb/Spry2/Bdnf/Gpr4/Gal/Nfkbia/Gli1/Ngf/Nfkb1/Arid5a/Inhba/Nrg1/Icam1/Cxcl10/Pdgfa/Lpar3/Cx3cl1/Hbegf/Sema4c/Akap6/Gadd45b/Ccl2/St5/Pvr/Icosl/Prr7/Gbp5/Cd40/Ptpn2/Rgs9/Bmp2/Map3k6/Ubd/Pdgfb/Pim2/Dnajc27/Rgs4/Ptgs2os
GO:0008283	cell proliferation	86/296	1,13E-07	Ifng/Il3/Nr4a3/Csf2/Tslp/Il5/Il6/March7/Nr4a1/Lif/Il11/Ptgs2/Serpine1/Pthlh/Cxcl1/Ereg/Il23a/Ripk2/Thbs1/Csf3/Foxc2/Adamts1/Egln3/Tnfaip3/Pgf/Vcam1/Tnfsf11/Atf3/Odc1/Has2/Ctla4/Dusp1/Stc1/Sox9/Areg/Dll1/Nod2/Ccl11/Twist2/Xirp1/Hilpda/Ift122/Tnc/Bmp6/Sox7/Cflar/Grem1/Egr1/Bdkrb2/Zfp36/Klf4/Spry2/Bdnf/Fosl2/Lrrc32/Gal/Nfkbia/Gli1/Ngf/Vash1/Ddit4/Inhba/Plau/Cxcl10/Pdgfa/Tnfsf9/Cx3cl1/Nos2/Hbegf/Junb/Marcksl1/Ptprn/Ccl2/Fst/Ovol1/Icosl/Nr5a2/Cd40/Ptpn2/Irs2/Bmp2/Pdgfb/Il2ra/Pim2/Fosl1/Tbx3
GO:0009888	tissue development	84/296	6,48E-08	Ifng/Nr4a3/Fgf23/Apold1/Il17f/Il6/Nr4a1/Popdc2/Lif/Ptgs2/Serpine1/Pthlh/Duox2/Thbs1/Foxc2/Ccno/Rhob/Pgf/Vcam1/Tnfsf11/Atf3/Nrap/Sik1/Actn2/Has2/Fos/Adamts9/Klf2/Stc1/Sox9/Trpv4/Areg/Myo18b/Xirp2/Dll1/Ccl11/Kdm6b/Myh6/Xirp1/Slc25a25/Ift122/Tnc/Mmp13/Bmp6/Sox7/Ryr2/Cflar/Grem1/Egr1/Tnnt2/Zfp36/Ppp3r1/Klf4/Spry2/Bdnf/Fosl2/Gpr4/Gal/Gli1/Ttn/Arid5a/Inhba/Plau/Nrg1/Icam1/Maff/Cxcl10/Pdgfa/Hbegf/Sema4c/Slc4a7/Junb/Akap6/Fst/Ovol1/Nr5a2/Rapgef3/Bmp2/Pdgfb/Bcor/Chd7/Rgs4/Yy1/Tbx3
GO:0042127	regulation of cell proliferation	80/296	2,02E-08	Ifng/Il3/Nr4a3/Csf2/Tslp/Il5/Il6/March7/Nr4a1/Lif/Il11/Ptgs2/Serpine1/Pthlh/Cxcl1/Ereg/Il23a/Ripk2/Thbs1/Csf3/Adamts1/Egln3/Tnfaip3/Pgf/Vcam1/Atf3/Odc1/Has2/Ctla4/Dusp1/Sox9/Areg/Dll1/Nod2/Ccl11/Twist2/Xirp1/Hilpda/Ift122/Tnc/Bmp6/Sox7/Cflar/Grem1/Egr1/Bdkrb2/Zfp36/Klf4/Spry2/Bdnf/Fosl2/Lrrc32/Gal/Nfkbia/Gli1/Ngf/Vash1/Inhba/Plau/Cxcl10/Pdgfa/Tnfsf9/Cx3cl1/Nos2/Hbegf/Marcksl1/Ptprn/Ccl2/Ovol1/Icosl/Nr5a2/Cd40/Ptpn2/Irs2/Bmp2/Pdgfb/Il2ra/Pim2/Fosl1/Tbx3
GO:0006468	protein phosphorylation	79/296	9,32E-07	Ifng/Il3/Fgf23/Csf2/Tslp/Il5/Il6/Akap12/Tnfsf15/Lif/Il11/Ptgs2/Cemip/Ereg/Il23a/Ripk2/Thbs1/Csf3/Ccno/Tnfaip3/Pgf/Tnfsf11/Atf3/Gdf15/Sik1/Dusp1/Sox9/Trpv4/Dusp8/Areg/Nod2/Ccl11/Obscn/Dusp5/Tnip1/Rell1/Bmp6/Ppp1r15a/Cflar/Prkcg/Grem1/Egr1/Inhbb/Bdkrb2/Zfp36/St3gal1/Klf4/Spry2/Bdnf/Spry4/Ttn/Ngf/Cdk5r1/Ddit4/Inhba/Plk2/Nrg1/Icam1/Pdgfa/Lpar3/Cx3cl1/Alpk2/Hbegf/Sema4c/Akap6/Gadd45b/Ccl2/St5/Cdk17/Cd40/Ptpn2/Rapgef3/Bmp2/Map3k6/Pdgfb/Plcl1/Pim2/Dnajc27/Sh2d3c
GO:0031399	regulation of protein modification process	78/296	6,52E-07	Ifng/Il3/Fgf23/Csf2/Tslp/Il5/Il6/Akap12/March7/Tnfsf15/Lif/Il11/Ptgs2/Cemip/Ereg/Il23a/Ripk2/Thbs1/Csf3/Ccno/Arrdc4/Tnfaip3/Pgf/Jdp2/Tnfsf11/Atf3/Gdf15/Dusp1/Sox9/Trpv4/Dusp8/Areg/Nod2/Ccl11/Dusp5/Tnip1/Rell1/Bmp6/Ppp1r15a/Cflar/Prkcg/Grem1/Egr1/Inhbb/Bdkrb2/Klf4/Spry2/Bdnf/Spry4/Ttn/Ngf/Cdk5r1/Arid5a/Ddit4/Inhba/Nrg1/Icam1/Pdgfa/Lpar3/Cx3cl1/Hbegf/Sema4c/Akap6/Gadd45b/Ccl2/Birc3/St5/Prr7/Cd40/Ptpn2/Rapgef3/Bmp2/Map3k6/Pdgfb/Bcor/Plcl1/Dnajc27/Sh2d3c
GO:0019220	regulation of phosphate metabolic process	74/296	1,49E-06	Ifng/Il3/Fgf23/Csf2/Tslp/Il5/Il6/Akap12/Tnfsf15/Lif/Il11/Ptgs2/Cemip/Ereg/Il23a/Ripk2/Thbs1/Csf3/Ccno/Ier3/Tnfaip3/Pgf/Tnfsf11/Atf3/Gdf15/Dusp1/Sox9/Trpv4/Dusp8/Areg/Nod2/Ccl11/Dusp5/Tnip1/Rell1/Bmp6/Ppp1r15a/Cflar/Grem1/Egr1/Inhbb/Bdkrb2/Klf4/Spry2/Bdnf/Spry4/Ttn/Ngf/Cdk5r1/Ddit4/Inhba/Nrg1/Icam1/Pdgfa/Lpar3/Cx3cl1/Nos2/Hbegf/Sema4c/Akap6/Gadd45b/Ccl2/Birc3/St5/Cd40/Ptpn2/Rapgef3/Irs2/Bmp2/Map3k6/Pdgfb/Plcl1/Dnajc27/Sh2d3c
GO:0042325	regulation of phosphorylation	73/296	2,27E-07	Ifng/Il3/Fgf23/Csf2/Tslp/Il5/Il6/Akap12/Tnfsf15/Lif/Il11/Ptgs2/Cemip/Ereg/Il23a/Ripk2/Thbs1/Csf3/Ccno/Ier3/Tnfaip3/Pgf/Tnfsf11/Atf3/Gdf15/Dusp1/Sox9/Trpv4/Dusp8/Areg/Nod2/Ccl11/Dusp5/Tnip1/Rell1/Bmp6/Ppp1r15a/Cflar/Grem1/Egr1/Inhbb/Bdkrb2/Klf4/Spry2/Bdnf/Spry4/Ttn/Ngf/Cdk5r1/Ddit4/Inhba/Nrg1/Icam1/Pdgfa/Lpar3/Cx3cl1/Hbegf/Sema4c/Akap6/Gadd45b/Ccl2/Birc3/St5/Cd40/Ptpn2/Rapgef3/Irs2/Bmp2/Map3k6/Pdgfb/Plcl1/Dnajc27/Sh2d3c
GO:0001932	regulation of protein phosphorylation	70/296	1,13E-07	Ifng/Il3/Fgf23/Csf2/Tslp/Il5/Il6/Akap12/Tnfsf15/Lif/Il11/Ptgs2/Cemip/Ereg/Il23a/Ripk2/Thbs1/Csf3/Ccno/Tnfaip3/Pgf/Tnfsf11/Atf3/Gdf15/Dusp1/Sox9/Trpv4/Dusp8/Areg/Nod2/Ccl11/Dusp5/Tnip1/Rell1/Bmp6/Ppp1r15a/Cflar/Grem1/Egr1/Inhbb/Bdkrb2/Klf4/Spry2/Bdnf/Spry4/Ttn/Ngf/Cdk5r1/Ddit4/Inhba/Nrg1/Icam1/Pdgfa/Lpar3/Cx3cl1/Hbegf/Sema4c/Akap6/Gadd45b/Ccl2/St5/Cd40/Ptpn2/Rapgef3/Bmp2/Map3k6/Pdgfb/Plcl1/Dnajc27/Sh2d3c

GO, gene ontology biological process terms. Genes showing differential expression (FC>1.5, adjusted p-value ≤ 0.05) were used as input for the analysis.
